# Brain volume abnormalities and clinical outcomes following paediatric traumatic brain injury

**DOI:** 10.1093/brain/awac130

**Published:** 2022-06-27

**Authors:** Niall J Bourke, Célia Demarchi, Sara De Simoni, Ravjeet Samra, Maneesh C Patel, Adam Kuczynski, Quen Mok, Neil Wimalasundera, Fareneh Vargha-Khadem, David J Sharp

**Affiliations:** Department of Brain Sciences, Imperial College London, London, UK; UK Dementia Research Institute, Care Research and Technology Centre, Imperial College London, London, UK; Department of Brain Sciences, Imperial College London, London, UK; UK Dementia Research Institute, Care Research and Technology Centre, Imperial College London, London, UK; Clinical Neuropsychology, Department of Psychological Services, Great Ormond Street Hospital, London, UK; King’s College London, Department of Psychology, Institute of Psychiatry Psychology and Neuroscience, De Crespigny Park, London SE5 8AF, UK; Department of Brain Sciences, Imperial College London, London, UK; Imaging Department, Imperial College Healthcare NHS Trust, Charing Cross Hospital, London W6 8RF, UK; Clinical Neuropsychology, Department of Psychological Services, Great Ormond Street Hospital, London, UK; Department of Paediatric Critical Care, UCL Great Ormond Street Institute of Child Health, London, UK; Paediatric Rehabilitation, Royal Children’s Hospital, Melbourne, Australia; Cognitive Neuroscience and Neuropsychiatry, UCL Great Ormond Street Institute of Child Health, London, UK; Department of Brain Sciences, Imperial College London, London, UK; UK Dementia Research Institute, Care Research and Technology Centre, Imperial College London, London, UK

**Keywords:** paediatric, traumatic brain injury, volume, developmental, cognition

## Abstract

Long-term outcomes are difficult to predict after paediatric traumatic brain injury. The presence or absence of focal brain injuries often do not explain cognitive, emotional and behavioural disabilities that are common and disabling. In adults, traumatic brain injury produces progressive brain atrophy that can be accurately measured and is associated with cognitive decline. However, the effect of paediatric traumatic brain injury on brain volumes is more challenging to measure because of its interaction with normal brain development. Here we report a robust approach to the individualized estimation of brain volume following paediatric traumatic brain injury and investigate its relationship to clinical outcomes.

We first used a large healthy control dataset (*n* > 1200, age 8–22) to describe the healthy development of white and grey matter regions through adolescence. Individual estimates of grey and white matter regional volume were then generated for a group of moderate/severe traumatic brain injury patients injured in childhood (*n* = 39, mean age 13.53 ± 1.76, median time since injury = 14 months, range 4–168 months) by comparing brain volumes in patients to age-matched controls. Patients were individually classified as having low or normal brain volume. Neuropsychological and neuropsychiatric outcomes were assessed using standardized testing and parent/carer assessments.

Relative to head size, grey matter regions decreased in volume during normal adolescence development whereas white matter tracts increased in volume. Traumatic brain injury disrupted healthy brain development, producing reductions in both grey and white matter brain volumes after correcting for age. Of the 39 patients investigated, 11 (28%) had at least one white matter tract with reduced volume and seven (18%) at least one area of grey matter with reduced volume. Those classified as having low brain volume had slower processing speed compared to healthy controls, emotional impairments, higher levels of apathy, increased anger and learning difficulties. In contrast, the presence of focal brain injury and microbleeds were not associated with an increased risk of these clinical impairments.

In summary, we show how brain volume abnormalities after paediatric traumatic brain injury can be robustly calculated from individual T_1_ MRI using a large normative dataset that allows the effects of healthy brain development to be controlled for. Using this approach, we show that volumetric abnormalities are common after moderate/severe traumatic brain injury in both grey and white matter regions, and are associated with higher levels of cognitive, emotional and behavioural abnormalities that are common after paediatric traumatic brain injury.

## Teaser

Bourke *et al.* identify paediatric brain injury patients with low brain volume for their age and associated cognitive difficulties by generating age norms from over 1200 healthy paediatric controls. Age matching is a robust way to overcome interpretation challenges related to healthy brain development during this period.

## Introduction

Paediatric traumatic brain injury (TBI) is common, affecting between 42 and 280 per 100 000 children.^1^ It often causes long-term disability, although outcomes are difficult to predict. Persistent cognitive, behavioural and psychiatric problems are common, including impairments of memory, attention, executive function and emotional control. These can have a major impact on academic performance and quality of life.^2^ The impact of TBI evolves over many years and can interact in complex ways with normal childhood development.^3^ However, the remote effects of TBI can be overlooked as developmental changes can obscure the persistent effects of brain injuries.^4^ A failure to recognize the long-term effects of paediatric TBI is problematic as it can limit a child’s access to appropriate healthcare and educational programmes, exacerbating the long-term effects of the injury.^2^ Hence, accurate methods to identify the degree and pattern of any significant brain injury are essential.

Neuroimaging is the main diagnostic tool for the evaluation of TBI. Brain injuries such as contusion and haemorrhage are usually readily apparent on routinely collected clinical imaging from CT and MRI, which is often acquired subacutely.^5,6^ These initial investigations provide useful and accurate information about the location and extent of many types of focal brain injury. However, diffuse injuries are not always apparent on standard imaging. This is particularly true for diffuse axonal injury, which is commonly produced by significant head injuries but is often missed on standard neuroimaging.^5,6^ Diffuse axonal injury is a key determinant of long-term clinical outcomes after TBI and can trigger progressive neurodegeneration. This may not be apparent on standard neuroimaging but can be assessed by measuring brain volumes using MRI.^7–9^ In adults, post-traumatic volume reductions in grey and white matter regions are seen in cross-sectional and longitudinal studies, showing that brain atrophy also progresses for many years after TBI.^7,9–12^ This post-traumatic atrophy in adults is associated with cognitive impairment, such as reduced memory performance and behavioural problems.^7,10^

Paediatric TBI can produce significant abnormalities in grey and white matter brain volume.^13^ Abnormalities in a range of brain regions have been reported in groups of paediatric TBI patients. Reductions in corpus callosum volumes are a relatively consistent finding in cross-sectional and longitudinal studies, which is often associated with ventricular enlargement.^14–16^ Limbic structures such as the hippocampus and amygdala are also commonly affected.^14,17,18^ Abnormalities in brain volume following paediatric TBI are associated with cognitive impairment.^14^ For example, reductions of cortical thickness in the medial frontal lobe and anterior cingulate are associated with impairments of emotional control and behavioural regulation.^19^ Working memory performance is associated with reductions in brainstem volume,^20^ and diffuse volume loss has been associated with reductions in processing speed, working memory and learning.^14^

Despite clear evidence supporting the value of measuring brain volumes in the assessment of TBI, this approach is not widely used clinically. Studies have generally focused on the analysis of patient groups and have not attempted to classify individual patients in relation to brain volumes. A key challenge is to define analysis methods that are robust to the challenges of clinical imaging data and that can be used to provide diagnostic information at the level of individual patients. This is particularly challenging in paediatric TBI as large developmental brain changes are associated with heterogenous and non-linear effects on brain volumes. Grey matter volumes reduce relative to head size during adolescence, whereas white matter volumes increase, making it critical to carefully account for age when interpreting brain volumes in children.^21,22^ In addition, progressive neurodegeneration triggered by TBI in children may be obscured by the normal relative reductions in grey matter volume seen during adolescence.

Here we present a pipeline for the volumetric analysis of paediatric MRI that is robust to common methodological challenges, including the impact of focal lesions and the impact of normal brain development. Using a large normative dataset of >1200 volumetric T_1_ MRI scans taken from freely available population data we first derive age curves for brain volumes in a range of white matter and grey matter regions. This provides individually age-matched normative control groups allowing a calculation of brain volume abnormalities that is robust to developmental effects. Using this approach, we classify individual paediatric TBI patients as having abnormal grey and white matter brain volumes and investigate whether cognitive, behavioural and emotional impairments seen after paediatric TBI are more common in patients with volumetric abnormalities.

## Materials and methods

### Population data

Healthy adolescent data was collated from publicly available repositories: The Human Connectome Project-Development (HCP-D), Autism Brain Imaging Data Exchange 1 & 2 (ABIDE 1&2).^23–25^ Participants were screened for exclusion criteria such as pre-existing psychiatric or neurological conditions according to local study protocols (*n* =  1232; mean age = 12.7; age range =  8–22; males = 852; females = 520). Data sharing agreements were confirmed for each repository as well as the current study.

### Local study participants

Thirty-nine patients with moderate-severe TBI (25 male, mean age ± SD = 13.53 ± 1.76) and 20 age-matched controls (10 male, mean age ± SD = 13.26 ± 2.21) were recruited and scanned at the Clinical Imaging Facility, Hammersmith Hospital, Imperial College London. To match for demographics many of these controls were siblings or related to the patient sample. Additional controls were recruited via poster and email notification at Great Ormond Street Hospital. Patients were recruited through specialist TBI outpatient clinics in London or referred from their local brain injury service on the basis of on-going functional and/or cognitive impairment. Severity of injury was based on the Mayo classification system.^26^ This considers duration of loss of consciousness, post-traumatic amnesia, lowest recorded Glasgow coma scale and neuroimaging (Supplementary Tables 1 and 2). Premorbid psychiatric and neurological illnesses were exclusion criteria, along with contraindication to MRI. The study was approved by London Riverside Research Ethics Committee (16/LO/1879). All participants under the age of 18 provided informed assent, with parents or guardians providing informed consent. A consultant neuroradiologist (author M.C.P.) reviewed all structural MRI scans, providing detailed clinical reports that included statements about the location of focal lesions and microbleeds.

### Neuropsychological assessment

All participants completed a detailed clinical neuropsychology battery, administered by a qualified Clinical Psychologist (author C.D.). Cognitive domains commonly associated with dysfunction following TBI were selected based on previous work and clinical need.^27–30^ Testing lasted between 3–4 hours and included measures of general intellectual abilities,^31^ verbal and non-verbal memory,^32–34^ attention, executive functions and speed of information processing,^35–38^ and academic attainment.^39^ Assessments of everyday functioning were collected through self and carer reports. These include self-assessment of wellbeing measured by the BECK Youth Inventories (BYI2),^40^ fatigue measured with the Paediatric Quality of Life Inventory (PedsQL)^41^ and apathy with the Lille Apathy Rating Scale (LARS).^42^ Self-report measures of wellbeing from the BYI2 include anxiety, depression and anger. Cognitive fatigue was measured with the PedsQL and Apathy was measured with the total summary measure from the LARS.

Carer reports of learning difficulties and adaptive functioning were measured with the Adaptive Behaviour Assessment System (ABAS),^43^ symptoms of attention-deficit/hyperactivity disorder were measured with the Conners-3,^44^ executive functioning with the Behaviour Rating Inventory of Executive Function (BRIEF2),^45^ mental health with the Strengths and Difficulties Questionnaire (SDQ)^46^ and fatigue with the PedsQL.^41^ Specific measures from carer reporting include learning difficulties from the ABAS, with subscales of adaptive functioning, communication, social skills and conceptual understanding investigated. Inattention, hyperactivity/impulsivity, learning difficulties were assessed with the Conners-3.^44^ The global executive composite was taken from carer rating from the BRIEF2. Overall stress was taken as a measure of wellbeing from carer rating from the SDQ. Cognitive fatigue was also measured with a carer rating from the PedsQL.

### Data reduction of neuropsychological assessment for group comparison

All neuropsychological measures were standardized for age and sex using population norms provided in the test manuals. A two-step data-driven approach was taken as a heuristic to select a reduced number of measures from a comprehensive neuropsychological battery for group comparison to reduce multiple comparisons. Step 1 involved an exploratory factor analysis to identify the broad components into which tasks with shared variance fell. Standardized scores, corrected for age and gender, were included in the analysis with a loading cut-off set at 0.3. In step 2, for each of the cognitive domains derived, tasks with the highest loading for each component in a 5-factor solution were selected for further group analysis.

### Hammersmith data MRI acquisition

MRI was performed on a Siemens MAGNETOM Verio 3.0 T scanner (Siemens Healthcare), using a 32-channel head coil. Each patient had conventional structural imaging, acquired with the following parameters: T_1_ MPRAGE [echo time (TE) = 2.98 ms, repetition time (TR) = 2.3 s, 1 mm isotropic voxel, 256 × 256 mm field of view, inversion time (TI) = 900 ms, flip angle = 9°, GRAPPA = 2, 5 min scanning time], T_2_ FLAIR (TE = 395 ms, TR = 5 s, TI = 1800 ms, 1 mm isotropic voxel, 250 × 250 mm field of view, GRAPPA = 2, 6 min scanning time), susceptibility weighted imaging (SWI) (120 1.2-mm-thick transverse slices, TR = 28 ms, TE = 20 ms, flip angle = 15°, in-plane resolution = 0.8 × 0.6 mm, field of view = 225 × 225 mm). Brain tissue volumes, white matter, grey matter and total intracranial volume (TICV) were computed for all participants, using a standard morphometry pipeline on T_1_-weighted images with SPM12, University College London, www.fil.ion.ucl.ac.uk/spm (1 October 2021, date last accessed).^47^ These procedures were previously described in more detail.^7^ Analysis of behavioural tests and MRI volume estimates were conducted using the R statistical environment.^48^

### Lesion segmentation

Semi-automatic segmentation, using a bespoke in-house Interactive Image Segmentation Tool v.1.8 (ImSeg, BioMedIA, Department of Computing, Imperial College London) was conducted to delineate brain areas with focal lesions. The tool has been used in previous TBI work.^12,49–51^ Segmentation is based on an algorithm for geodesic image segmentation.^52^ T_1_-weighted and FLAIR images were imported into the software and coregistered to the T_1_ native space. Lesion maps were drawn as overlays on the T_1_-weighted images, adjusting the FLAIR opacity to better discern lesion boundaries. Areas of damaged tissue are manually labelled as such. The algorithm updates to fill in the surrounding areas of similarly damaged tissue. Multiple permutations to correct over/under estimations are required with final manual edits around lesion boundaries. Masks were drawn by author R.S. following training by author N.J.B. Lesions were reviewed by a consultant neuroradiologist M.C.P. Lesion probability distribution maps were generated from the individual participant binary lesion masks by transformation to MNI standard space using Advanced Normalization Tools.^53^

### Neuroimaging analysis workflow

#### Population cohort workflow

Healthy control data was gathered from Human Connectome Developmental (*n* = 644), ABIDE1 (*n* = 327) and ABIDE2 (*n* = 261) for a population volume dataset. Each of the datasets obtained to generate this population cohort followed standardized scanning procedures. Details of these protocols can be found.^23–25^ A standard voxel-based morphometry pipeline was implemented with SPM12, described previously.^7^ In brief, initial processing segmented brain volumes into white and grey matter. A study template was defined with DARTEL registration using a representative sample of healthy adolescent participants across all sites (*n* = 152). The template was affine registered to MNI152 space. Individual images were normalized to the study template. Steps were taken to control for data quality. A visual inspection of registration was performed during preprocessing. Global grey and white matter estimates of the processed data were plotted for each site. This revealed a site with abnormally low estimates that stemmed from low contrast and was subsequently removed. To control for extreme scores in the population data that may be a source of noise in the data *Z*-score capping was performed at (±3 SD). Assuming a normal distribution, this threshold retains over 99% of the data, only removing very unlikely estimates, resulting in a cleaned reference population cohort split by age (8–22) for further analysis with an independent adolescent dataset (Supplementary Fig. 1 and Supplementary Table 3). A representative sample across all sites from the QC population data were selected for an adolescent template (*n* = 152).

Global estimates and selected regions of interest were extracted from the population data for each age year to generate normative age range estimates. White matter region of interest selection was motivated by a previous diffusion pipeline identifying reliable white matter tracts with good anatomical coverage relating to a range of motor and cognitive impairment following TBI.^6^ These tracts include the subdivisions of the corpus callosum, the corticospinal tracts, corona radiata, inferior longitudinal fascicules and the middle cerebellar peduncle. Similarly, to avoid global mean estimate, specific grey matter regions of interest were selected covering key anatomical regions with association to cognitive function frequently affected following TBI. These grey matter regions of interest include the inferior frontal gyrus, anterior cingulate gyrus, posterior cingulate gyrus, anterior and posterior para-hippocampus, insula, amygdala, caudate, putamen and thalamus. Bilateral estimates were taken for all regions of interest.

#### Volume assessment in an independent local clinical dataset of healthy controls and TBI patients

**Figure 1:**
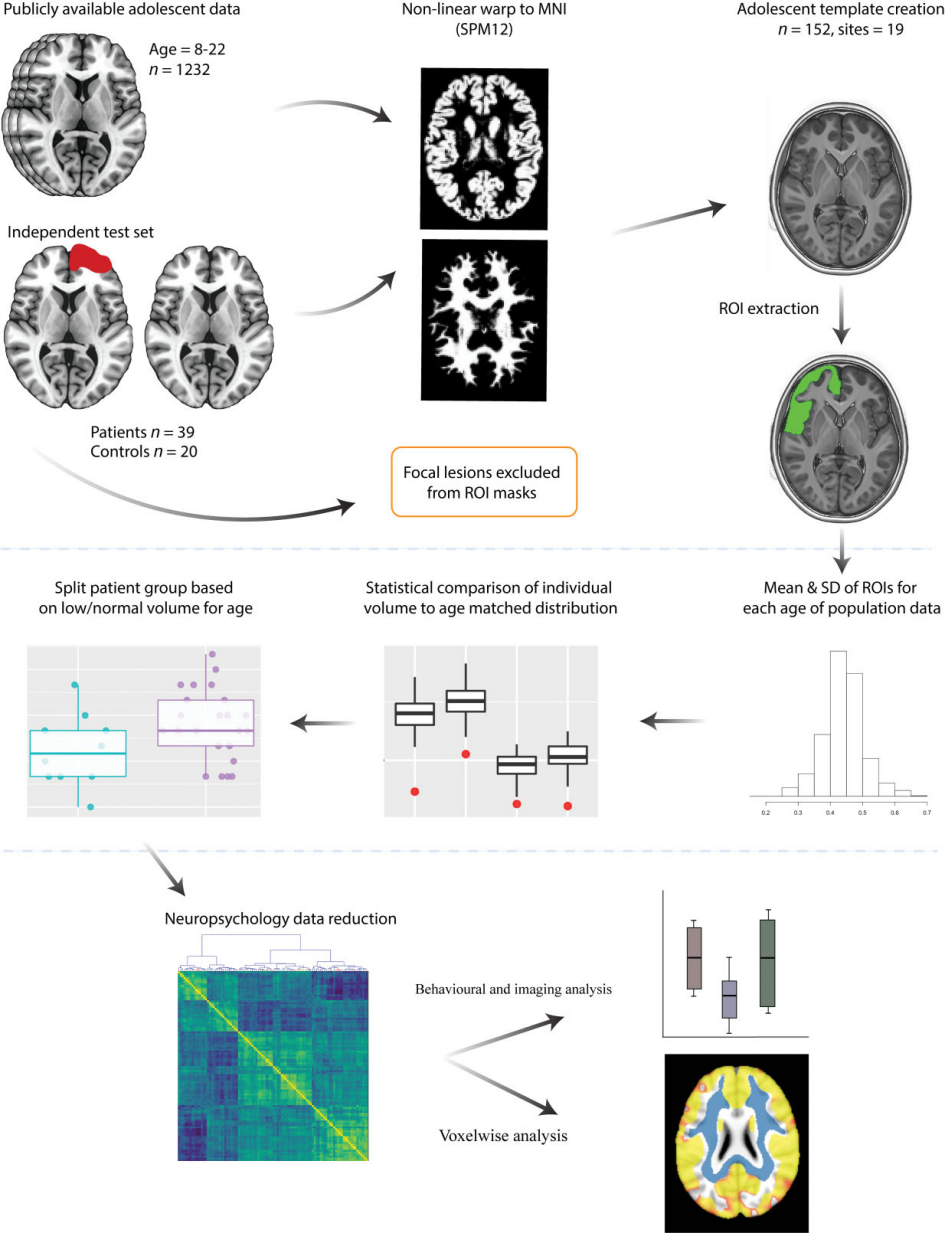
**Study workflow**. Population and study MRI data were preprocessed using a standard SPM12 VBM pipeline. A representative sample of participants were randomly selected across the 19-scanning site of the population data to create an adolescent template. Patients with focal lesions had masks drawn that were excluded from region of interest (ROI) estimates. For each region of interest means and standard deviations (SD) were calculated for age. Individual comparisons were performed against these normative estimates using an independent set of control and paediatric TBI data.

An independent clinical dataset comprising of 39 adolescent TBI patients and 20 healthy controls underwent the same preprocessing pipeline, using the previously generated adolescent template. Global and region of interest estimates were extracted and corrected for TICV. For each participant in the test dataset a *Z*-score was calculated based on the mean and standard deviation of the age-matched population data, corrected FDR multiple comparisons for the number of regions of interest included. This produces an individual assessment of volume in relation to an age-matched population. From here, the TBI group was subdivided into patients classified as having low or normal volume in relation to the age normed population (Fig. 1). It was proposed that patients classified as having abnormally low volume for their age would show greater impairment on neuropsychological assessments.

Thirteen patients with gross anatomical abnormalities due to presence of focal lesions had these regions masked during extraction of volume estimates for each region of interest. In addition, a unique control dataset was generated for each of these patients extracting volume estimates excluding their matching lesion regions to avoid the confound of focal lesions in classifying volume abnormalities. The same analysis pipeline was conducted as described previously. Subsidiary analysis indicated this approach did not produce significantly different results to comparing patients with focal lesions to the main population control cohort (Supplementary Fig. 2).

### Data availability

The Human Connectome Project-Development data used in this paper can be accessed through the National Institute of Mental Health Data Archive (https://nda.nih.gov/, last accessed 1 October 2021). The Autism Brain Imaging Data Exchange can be accessed via NITRC and INDI (http://fcon_1000.projects.nitrc.org/indi/abide/, last accessed 1 October 2021). The study site controls and paediatric brain injury can be obtained through authors with appropriate transfer agreements.

## Results

### Developmental changes in grey and white matter during healthy adolescence

**Figure 2:**
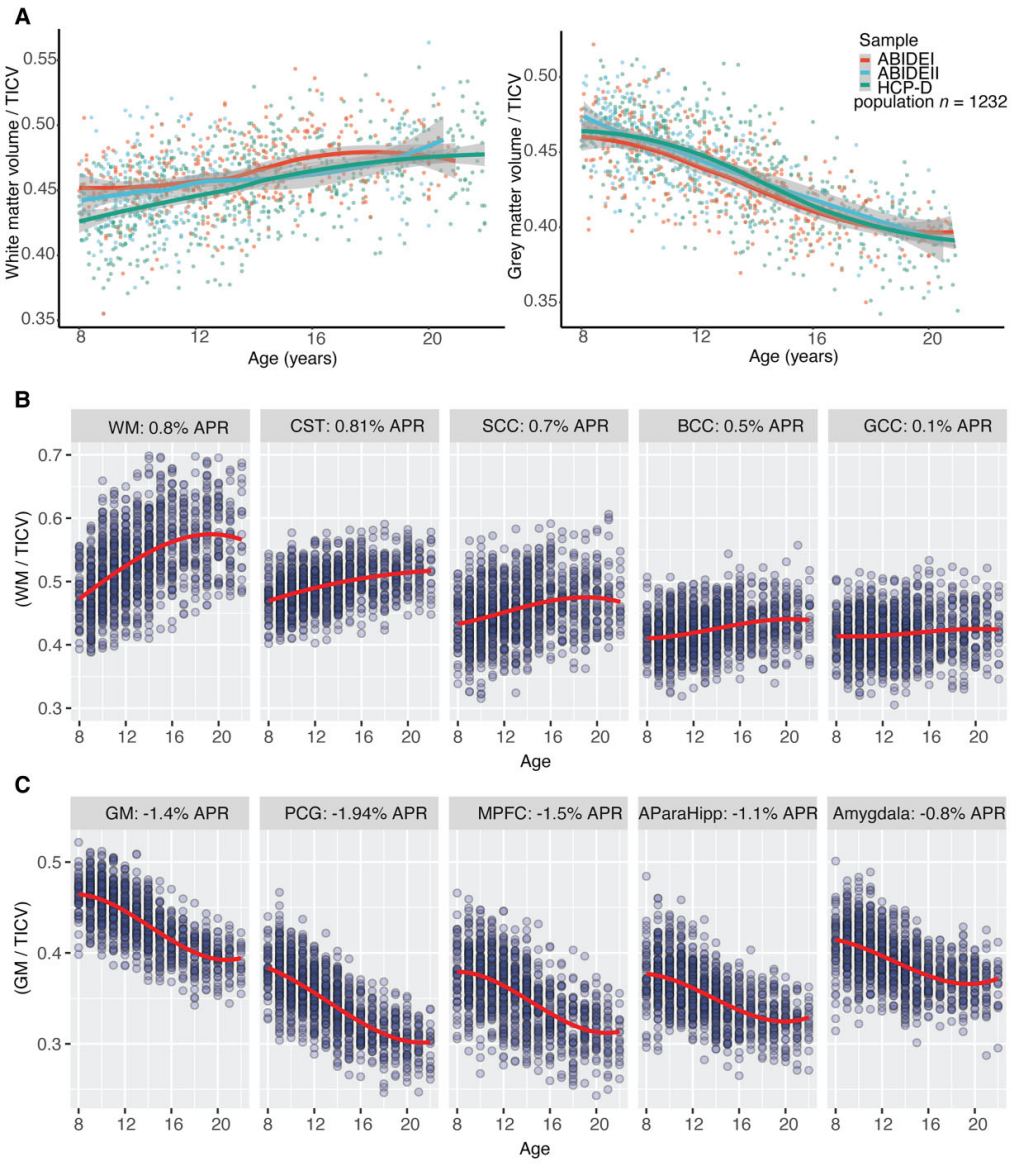
**Grey and white matter age curves for population and study cohorts, corrected for TICV**. (**A**) Age curves from 1232 individuals from the ABIDE 1, ABIDE 2 and HCP-D cohorts. (**B**) Selected white matter regions of interest highlighting regional variation in average annual percentage rate change (APR) of volume corrected for TICV. BCC = body of corpus callosum; CST = corticospinal tracts; GCC = genu of the corpus callosum; SCC = splenium of corpus callosum. (**C**) Selected grey matter region of interest of average APR change of volume corrected for TICV. AParaHipp = anterior para-hippocampus; MPFC = medial prefrontal cortex; PCG = posterior cingulate cortex.

Grey and white matter brain volumes change significantly through adolescence as the brain develops. Using >1200 volumetric T_1_ scans from three paediatric cohorts aged between 8 and 22 (HCP-D, ABIDE 1, ABID2) we calculated age-related changes in grey and white matter volumes through adolescence (Fig. 2A). As expected, TICV increased as individuals age (Supplementary Fig. 3). After correcting for this increase in head size, mean global white matter volume increased ∼9% between the ages 8 and 20, with rates varying across brain region. For example, the cortical spinal tract increased by ∼0.8% on average per year, whereas there were smaller rates of increase observed for the body of the corpus callosum (∼0.5% per year) and the genu (∼0.1% per year) (Fig. 2B). In contrast, mean global grey matter volume reduced 17% across the same period. Reductions in grey matter volume varied across brain regions. For example, the posterior cingulate gyrus (PCG) reduced by ∼1.9% on average per year between the age of 8 and 20, whereas the amygdala reduced by ∼0.8% on average per year (Fig. 2C). Rates of volume change for a range of grey matter and white matter regions are reported in Supplementary Figs 4 and 5. Age explained 46.3% of the variance in grey matter volumes, whereas sex explained <1% and cohort 1.5%. For white matter, age explained 25% of the variance, cohort 2% and sex <1%.

### Traumatic brain injury group

Our large normative dataset was then used to investigate the impact of paediatric TBI on brain volumes. We recruited 39 moderate-severe paediatric TBI patients and 20 age-matched healthy controls. MRI was acquired on the same scanner for paediatric TBI subjects and local controls. The mechanism of injuries varied across participants: road traffic accident (*n* = 20), assault (*n* = 1), falls (*n* = 11) and sport injuries (*n* = 4) accounted for most causes. Patients were investigated in the post-acute/chronic phase of injury (median time since injury 14 months, range 4–168 months). Median age at injury was 141 (53.14 months, range 2–191). Twenty-seven patients had a lowest reported GCS ≤ 12, nine of which had a lowest reported GCS of 3. Loss of consciousness ranged from 30 s to >3 h. Full clinical characteristics are reported in Supplementary Table 2.

### Neuropsychological and behavioural impairments

The paediatric TBI group showed a range of post-traumatic cognitive impairments (Supplementary Table 4). After multiple comparison correction, impairments were observed in intelligence quotient (IQ) (WASI-II), processing speed [Delis-Kaplan Executive Function System (DKEFS) 2,3,4, Trail making A and B] and memory [Children’s Memory Scale (CMS) immediate, delayed, recognition recall, the doors and people immediate and delayed recall and California Verbal Learning Test (CVLT) learning] (*P* < 0.05 for all). No significant group differences were seen in executive functioning and attention.

**Figure 3:**
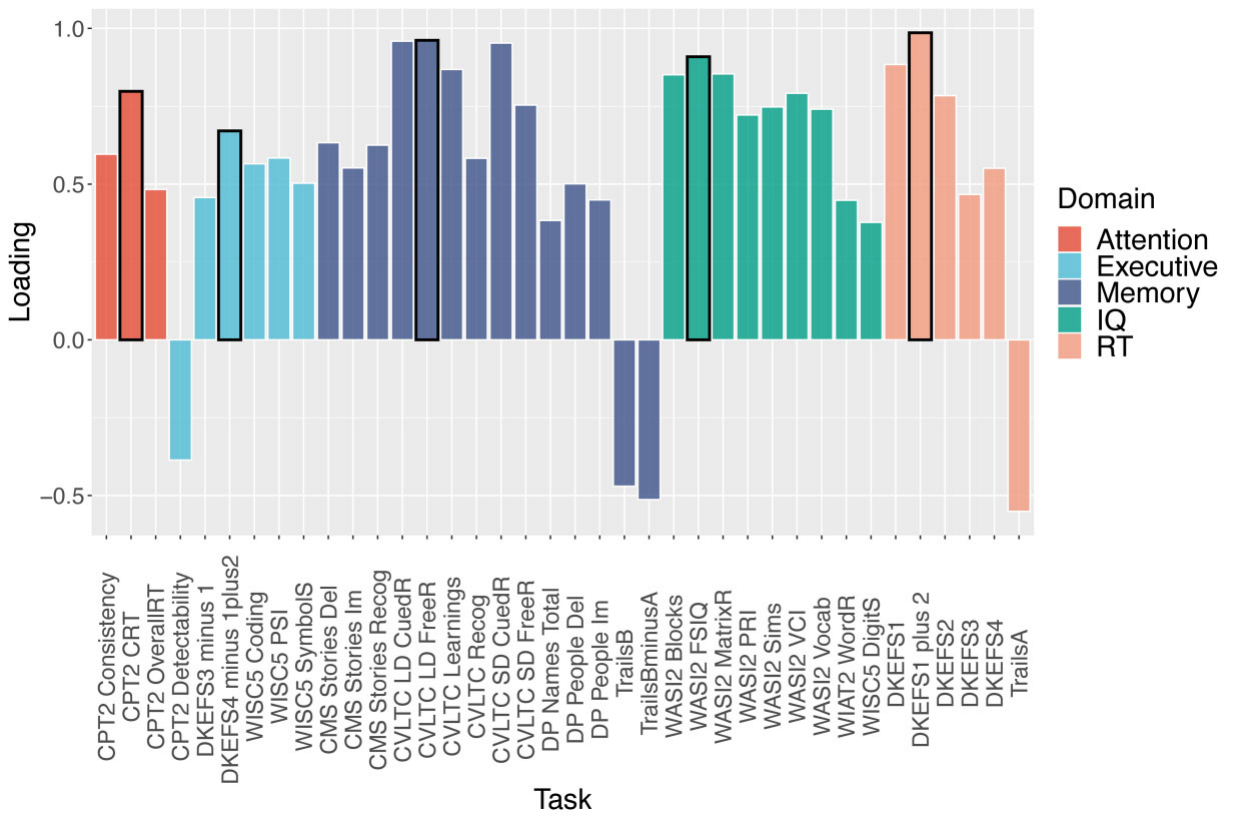
**Summary of exploratory factor analysis of neuropsychological measures**. Colour coding illustrates main factors. For each factor, the highest loading task is highlighted and selected as a representative measure for independent group comparisons. CMS = Children’s Memory Scale; CPT2 = Continuous Performance Task – 2; CVLT = California Verbal Learning Test; DKEFS = Delis-Kaplan Executive Function System; DP = Doors and People test; Trails = Trail making Test; WASI2 = Weschler Abbreviated Scale of Intelligence; WISC = Weschler Intelligence Scale for Children.

An exploratory factor analysis was used identify factors explaining the majority of individual variability in cognitive performance. Five factors explained 72% of the variance (Fig. 3). These were chosen based on a prior expectation of separable cognitive domains and the results of a scree plot of eigenvalues (Supplementary Fig. 6). Most tests loaded on one of these factors. Where tests loaded across factors, the highest loading was considered. Tests with the highest loadings were selected for further analysis, i.e. reaction time = DKEFS 1 + 2, IQ = Full-Scale Intelligence Quotient (FSIQ), executive = DKEFS 4 minus (1 + 2), memory = CVLT-C long delay free recall and sustained attention = CPT-II Change Reaction Time (Fig. 3 and Supplementary Table 6).

The paediatric TBI group also showed significant impairments in behaviour and psychiatric state as assessed by the patients and the carers (Supplementary Tables 4 and 5). Patients reported increases in anger [*t*(235) = 2.27, *P* = 0.02], fatigue [*t*(235) = 3.14, *P* = 0.002] and apathy [*t*(235) = 4.52, *P* < 0.001] compared to controls. Differences were not observed in anxiety or depression. Carers reported reductions in behavioural regulation [*t*(312) = 3.2, *P* = 0.001], emotional control [*t*(312) = 2.95, *P* = 0.003], working memory [*t*(312) = 3.9, *P* < 0.001] and impact on life [*t*(312) = 2.7, *P* = 0.005]. There was increased emotional distress [*t*(312) = 2.18, *P* = 0.03], fatigue [*t*(312) = 4.36, *P* < 0.001], executive functioning [*t*(312) = 3.6, *P* < 0.001]. Borderline increases in behavioural difficulties were reported [*t*(312) = 1.99, *P* = 0.047].

#### Focal brain injury and microbleeds do not explain cognitive or behavioural problems

**Figure 4:**
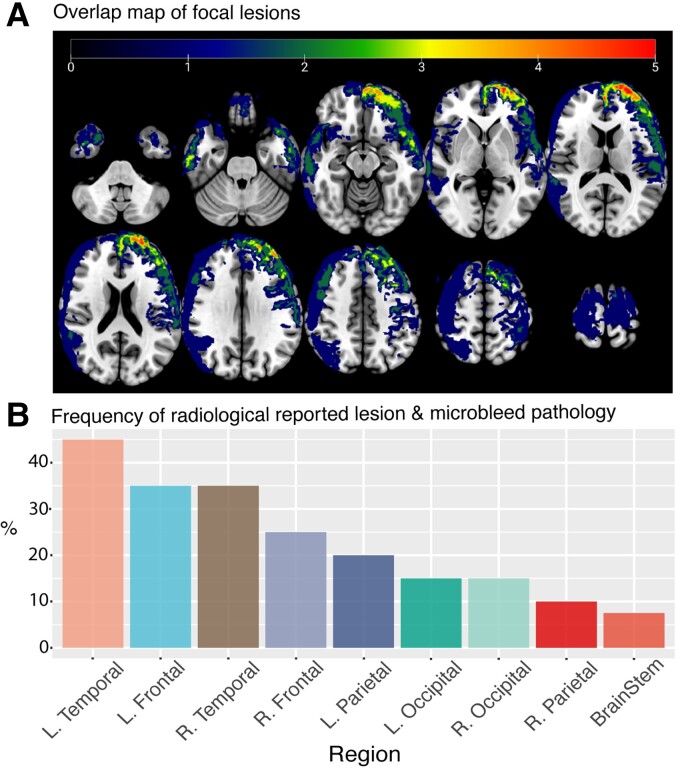
**Lesion distribution map and radiological reporting**. (**A**) Lesion overlap map of participants. Thirteen out of the 39 paediatric TBI patients tested in the study presented with focal lesions. These were regressed out of analysis on an individual bases. (**B**) Frequency of radiological reported focal pathology as percentage of patient sample (including focal lesions and traumatic microbleeds). L = left; R = right.

Thirteen TBI patients (33%) had evidence of focal damage defined on T_1_ and FLAIR imaging [median = 14 308 mm^3^ (range = 89–197 237 mm^3^)] (Fig. 4). This was most frequently seen bilaterally in the frontal and temporal poles. Microbleeds, a marker of vascular injury seen on SWI are a separable pathology to focal lesions and were present in 23 patients (59%), commonly in a parafalcine distribution with frontal (13, 33%), temporal (22, 56%), parietal (8, 20%) and occipital lobes locations (9, 23%), and additional microbleeds within the brainstem (3, 8%) and putamen (one patient). A negative relationship is seen between lesion volume (log transformed) and brain grey matter volume estimates, where larger lesion sizes are associated with lower brain volume (*r* = −0.78, *P* = 0.001) (Supplementary Fig. 7). However, patients with and without focal lesions did not have significant different brain volumes [grey matter (*t*(36) = 0.89, *P* = 0.37), white matter (*t*(36) = 1.05, *P* = 0.30)]. All further analysis of volume estimates control for presence of focal lesions as a nuisance regressor. The presence or absence of focal brain injuries or microbleeds did not explain individual differences on the five cognitive tasks described previously and behavioural impairments from carer and self-reports. Patients with and without focal injury (13 versus 26) or microbleeds (23 versus 16) did not show differences in the five selected cognitive tasks or in reports of everyday behavioural and psychiatric functioning (*P* > 0.1, FDR corrected for all measures).

### Grey and white matter volumes are reduced following TBI

**Figure 5:**
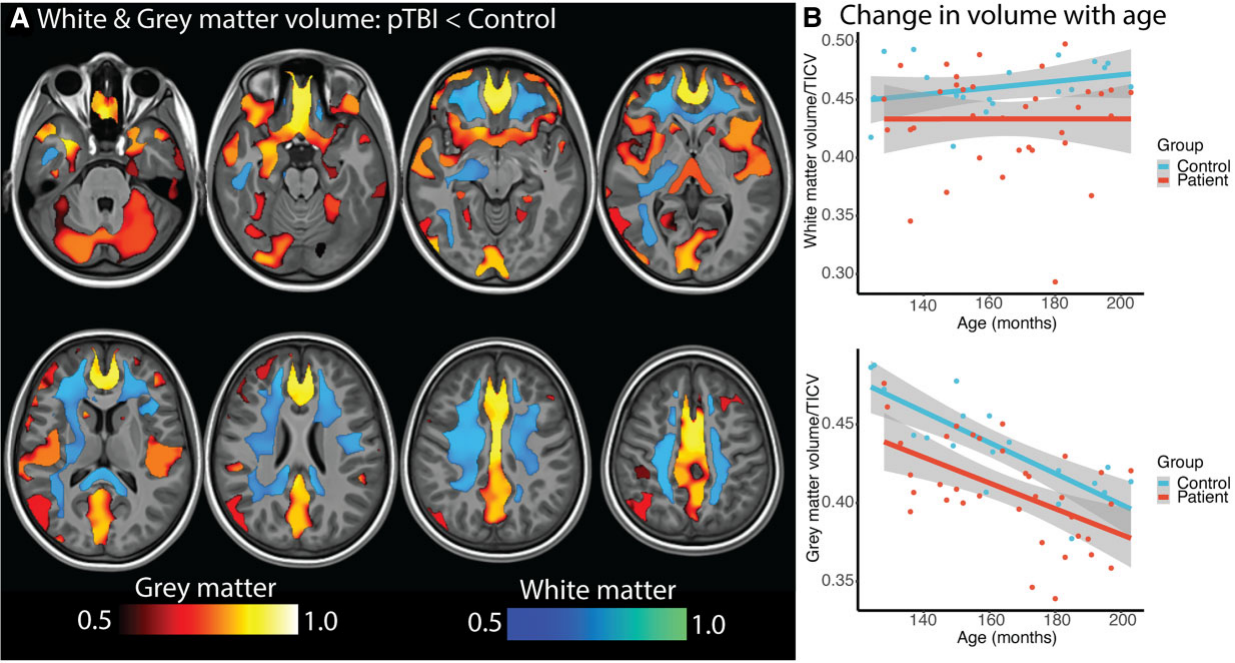
**Voxelwise volume analysis (local study participants)**. (**A**) Blue-green heat map = areas or reduced white matter in TBI patients compared to controls; red-yellow heat map = areas of reduced grey matter in TBI patients compared to controls. (**B**) Plot of TICV corrected grey matter and white matter with age in the Hammersmith study group. Adjusted for age, intracranial volume, voxelwise regression of lesion regions (threshold-free cluster enhancement: *P* < 0.05, corrected for multiple comparisons).

Paediatric TBI patients showed reduced grey and white matter volumes compared to both local controls (Fig. 5A) and our large cohort. Voxelwise analysis of the TBI group showed volume reductions in midline structures including the anterior and posterior cingulate cortices compared to local controls. Reduced cortical volumes were also seen in the insula, cerebellum and the prefrontal cortex. White matter volume reductions were also seen in a range of association fibres such as the superior longitudinal fasciculus and cingulum as well as midline tracts including the genu, splenium and body corpus callosum and corticospinal tracts.

Region of interest analysis was used to compare the paediatric TBI to our large control cohort. Grey matter volumes showed a significant main effect for group in grey matter [*F*(2,1344) = 19.42, *P* < 0.001] due to lower volume in patients compared to population controls. Grey matter volumes reduced with age [*F*(2,1344) = 1143, *P* < 0.001], with no interaction between group and age. Lower white matter volumes were seen in the TBI group [*F*(2,1344) = 21.18, *P* < 0.001], with age associated with increasing volume across both groups [*F*(2,1344) = 417.12, *P* < 0.001] and no interaction.

### Post-traumatic volume changes in individual patients

We next investigated volumetric changes produced by TBI at the individual level. Paediatric TBI patients were compared to population controls of the same age to assess any significant deviation from the normal range (*n* > 44 for each year of age) (Supplementary Table 3). Grey and white matter regions of interest were investigated using an approach developed for diffusion tensor imaging (DTI) analysis of TBI effects.^6^ Cortical regions, white matter tracts and whole grey/white matter volumes were investigated,^6^ with the presence of focal lesions controlled for by their removal from each region of interest investigated.

**Figure 6:**
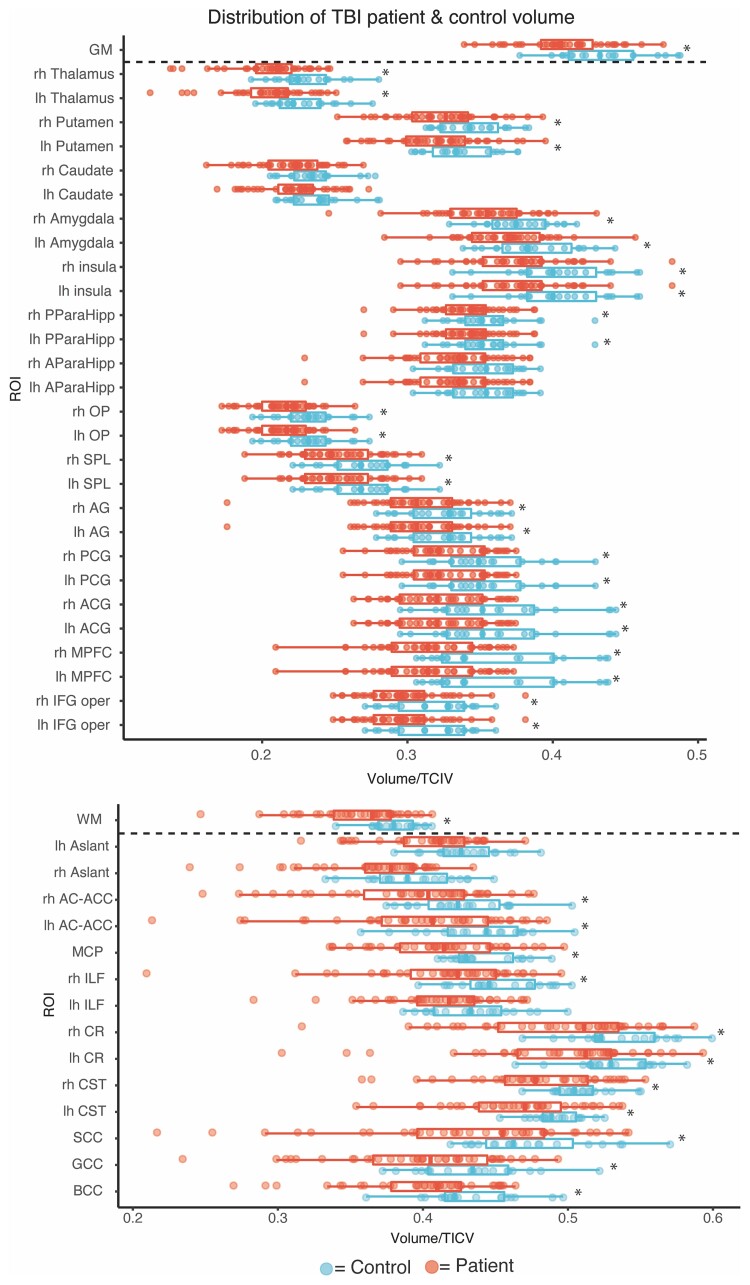
**Summary of region of interest volume for local study controls (blue) and PTBI patients (red) against population age norms (box plots)**. AC-ACC = anterior caudate – anterior cingulate cortex; ACG = anterior cingulate gyrus; AG = angular gyrus; AParaHipp = Anterior para hippocampus; BCC = body corpus callosum; CR = corona radiata; CST = cortical spinal tract; GCC = genu corpus callosum; GM = grey matter; IFG oper = inferior frontal gyrus opercularis; ILF = inferior longitudinal fasciculus; lh/rh = left/right hemisphere; MCP = medial cerebellar peduncle; MPFC = medial prefrontal cortex; OP = occipital pole; PCG = posterior cingulate gyrus; PParaHipp = posterior para hippocampus; ROI = region of interest; SCC = splenium corpus callosum; SPL = superior parietal lobule; WM = white matter.

White and grey matter volume abnormalities were common following paediatric TBI (Fig. 6, Supplementary Figs 8, 9 and Supplementary Table 7). Of the 39 patients, 11 (28%) were identified as having at least one white matter abnormality (i.e. low volume) compared to age-matched population norms after multiple comparison correction. Of these, five (56%) had abnormalities in four or more regions of interest. Seven patients (18%) were identified as having at least one grey matter region of interest abnormality (i.e. volume reductions). Of these, four (36%) had abnormalities in four or more regions of interest. Five (12%) patients were identified as having low whole brain white matter volume, three (7.5%) were identified as having low whole brain grey matter volume. Six (55%) of patients identified with regional low white matter volume did not have abnormalities in ‘global’ estimates of white matter. Similarly, three (43%) of patients with regional abnormalities in grey matter did not have global abnormalities. No local healthy controls were classified as abnormal using this approach when compared to the population controls.

Following classification of patients into normal and low volume subgroups using population data, we compared this patient subgrouping against our independent local study controls. Large effect size differences are seen across all regions of interest in the low volume group compared to local study controls, whereas no significant effects are seen in the normal volume patient group compared to controls (Supplementary Fig. 8). A summary of imaging classifications including acute CT, MRI radiology report and volume classification is included in Supplementary Table 8.

### Case studies

**Figure 7:**
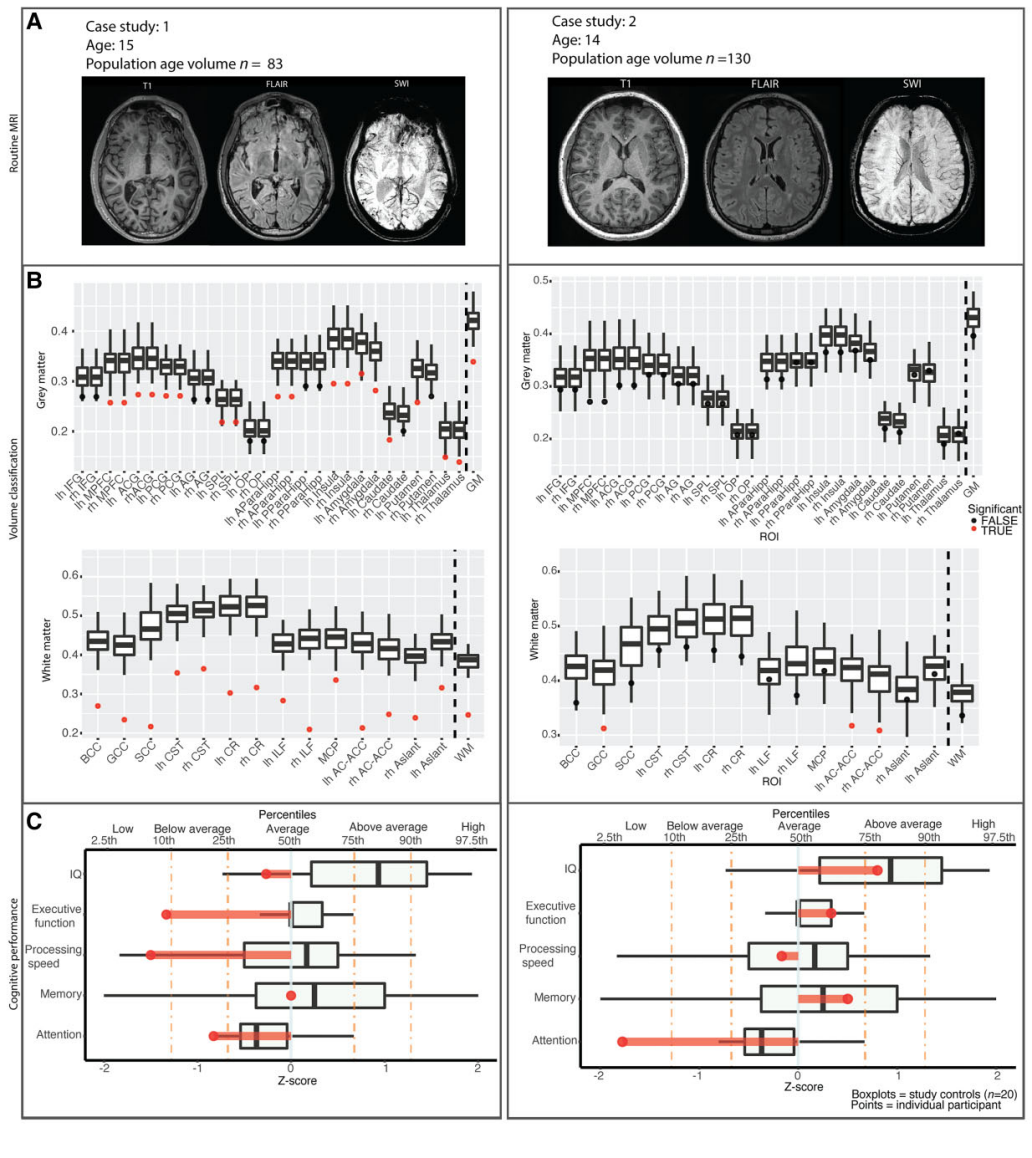
**Individual case studies illustrating images from routine MRI (A), individual volume classification against matching population norms (B) and cognitive performance (C).** Cognitive performance transformed into population standard scores with individual patients plotted against study controls. lh = left hemisphere, rh = right hemisphere, IFG = inferior frontal gyrus, MPFC = medial prefrontal cortex, ACG = anterior cingulate gyrus, PCG = posterior cingulate gyrus, SPL = superior parietal lobule, OP = occipital pole, BCC = body of corpus callosum, GCC = genu of corpus callosum, SCC = splenium of corpus callosum, CST = cortical spinal tract, CR = corona radiata, ILF = inferior longitudinal fasciculus, MCP = medial cerebellar peduncles, AC-ACC = anterior caudate-anterior cingulate cortex.

The value of the individual approach is illustrated by two case studies. The first was 13 years old when as a pedestrian she was hit by a car. She required a decompressive craniectomy due to a skull fracture and midline shift evidenced from initial CT (Fig. 7). She was assessed age 15, 36 months after injury and performed in the ‘low’ range for processing speed and executive function, with below average performance on attention as measured by normative scores on the Conners’ Continuous Performance Test II (Fig. 7C). Routinely clinically acquired MRI acquisitions including T_1_, FLAIR and SWI showed frontal and temporal cortical contusions and radiological evidence of diffuse axonal injury. Visual reporting identified low cortical volume for a person of this age. Our pipeline identified widespread low white matter and grey matter volume (Fig. 7B and C in both grey matter and white matter structures). This case shows evidence of severe TBI in standard neuroimaging, with congruent results from volumetric analysis.

Case study 2 was an 11-year-old cyclist when he was hit by a car travelling at ∼30 miles an hour. He was not wearing a helmet. The patient was intubated on scene and remained unconscious for 2 weeks. Initial CT indicated right frontal bone fracture but no focal brain injury. The patient was age 14 years old and was assessed 32 months after injury. In general, his intellectual abilities were in the high average range measured with the WASI-II and WISC-V with expected academic attainments in school. However, neuropsychological assessment showed selective low performance with attention. There were also persistent difficulties in managing anger and concerns about wellbeing and social communication skills. Standard MRI was reported as normal, however, quantitative volume analysis indicated low white matter volume in the genu of the corpus callosum and the tract connecting the anterior caudate to the anterior cingulate cortex, which we have previously reported to be damaged in patients with post-traumatic impairments of attention and executive function.^54^ This case illustrates the value of identifying subtle but clinical important abnormalities that were not identified with standard radiological reporting.

### Processing speed impairments in TBI patients with brain volume abnormalities

**Figure 8:**
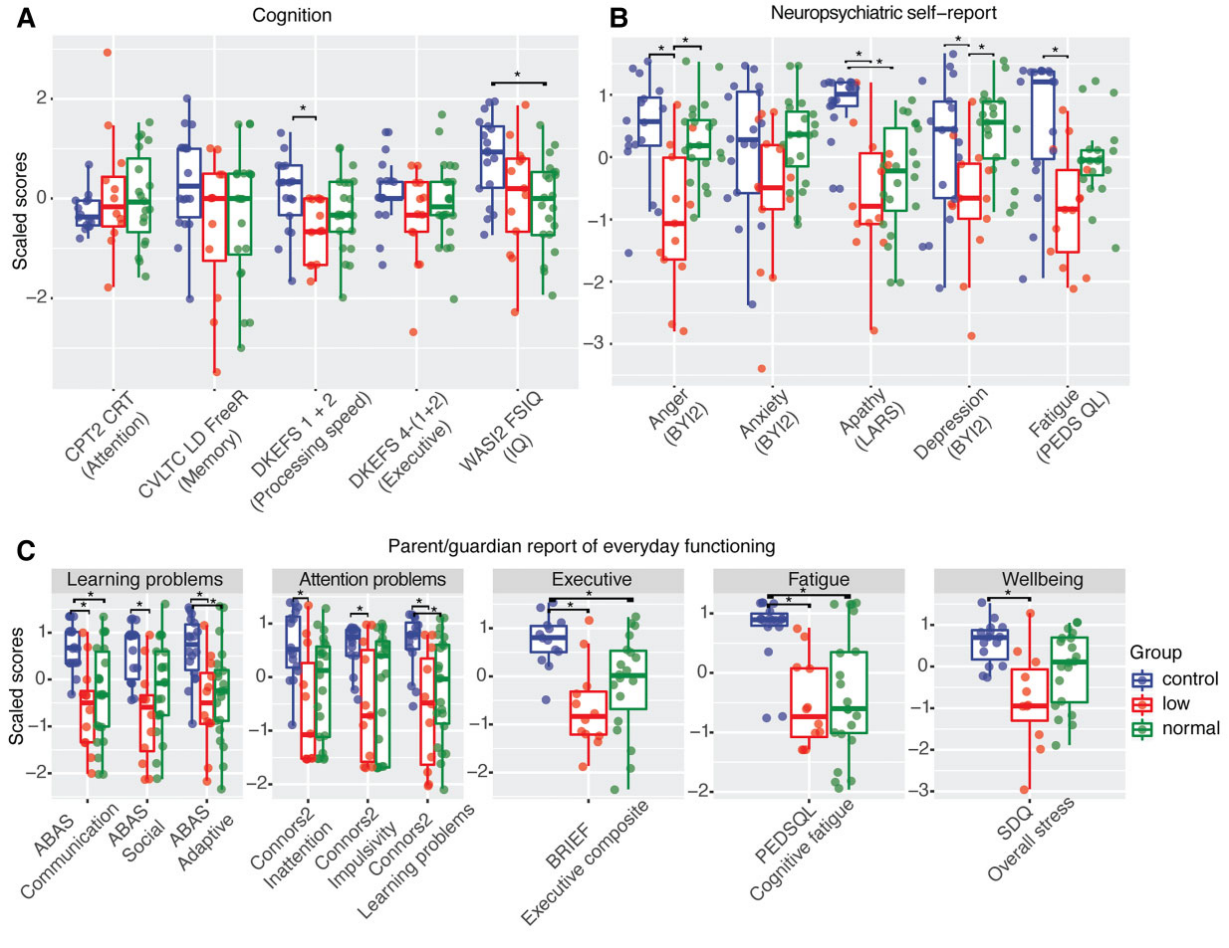
**Neuropsychology and behavioural assessment.** (**A**) Results of cognitive performance. (**B**) Self-report assessment of neuropsychiatric symptoms. (**C**) Carer reports of everyday functioning.

The relationship between cognitive performance and volume classification was investigated across the five cognitive tasks selected from the exploratory factor analysis. Patients were divided into low volume or normal volume groups based on whether any areas of low grey matter or white matter volume were identified. Volumetric abnormalities were associated with impairments in processing speed following paediatric TBI. One-way ANOVA revealed a main effect of group on processing speed [*F*(2,43) = 3.5, *P* = 0.03]. This was due to slower processing speed in the low volume group relative to controls [*t*(43) = 2.64, *P* = 0.03]. Intellectual functioning also showed a main effect of group [*F*(2,43) = 3.64, *P* = 0.03], the result of the normal volume group having lower intellectual functioning compared to controls [*t*(43) = 2.63, *P* = 0.03]. No other significant group associations were seen for attention, memory and executive functioning (*P* > 0.05) (Fig. 8).

### Neuropsychiatric function and brain volume abnormalities

Brain volumes were also related to the presence of neuropsychiatric symptoms and parental reports of injury impact. One-way ANOVA showed a main effect of group on self-reports of anger [*F*(2,47) = 11.46, *P* < 0.001], the result of increased anger in the low volume group relative to both controls [*t*(47) = −4.4, *P* < 0.001] and the normal volume TBI group [*t*(47) = −4.1, *P* < 0.001]. There was a main effect of depression [*F*(2,47) = 6.06, *P* < 0.01], the result of more depressive symptoms in the low volume group compared the normal volume TBI group [*t*(47) = −3.4, *P* < 0.001]. A main effect of group on cognitive fatigue was seen [*F*(2,47) = 9.73, *P* < 0.001], the result of more fatigue seen in the low volume compared to healthy control group [*t*(47) = −4.4, *P* < 0.001]. A main effect on apathy was also seen [*F*(2,47) = 14.29, *P* < 0.001]. Patients with low volume had higher levels of apathy compared to healthy controls [*t*(47) = −4.8, *P* < 0.001]. TBI patients with normal volume also had higher levels of apathy compared to healthy controls [*t*(47) = −4.4, *P* < 0.001]. No significant differences were seen for reported levels of anxiety (Fig. 8B).

Carer reports of post-traumatic problems also differed across the groups, although significant differences were generally seen between controls and both low and normal volume groups. Learning impairment was measured using the ABAS scale. One-way ANOVA of adaptive functioning measured using the ABAS showed group differences [*F*(2,48) = 7.72, *P* < 0.01]. TBI patients with low volume having more problems in adaptive functioning than controls [*t*(48) = −3.4, *P* < 0.01] and TBI patients with normal volume also having more problems than controls [*t*(48) = −3.2, *P* < 0.01]. Group differences were seen for social ability [*F*(2,48) = 8.68, *P* < 0.001]. The low volume group were rated as having poorer social ability compared to controls [*t*(48) = −4.1, *P* < 0.001]. Group differences were seen for communication [*F*(2,48) = 10.82, *P* < 0.001]. Greater communication problems in the low volume group were seen compared to controls [*t*(48) = −4.2, *P* < 0.001] and the normal volume group also had greater communication difficulties compared to controls [*t*(48) = −3.8, *P* < 0.001] (Fig. 8C).

Carer reports of attention problems measured by the Conners-3 scale showed group differences [*F*(2, 46) = 6.15, *P* < 0.01], the result of patients with low TBI volume having higher symptoms compared to controls for inattention [*t*(46) = −3.3, *P* < 0.01] and the normal volume group compared to controls [*t*(46) = −2.63, *P* = 0.03]. Group differences were seen on the impulsivity subscale [*F*(2,45) = 5.19, *P* < 0.01]. The result of higher impulsivity among low volume patients were seen compared to controls [*t*(45) = −2.98, *P* = 0.01] and normal volume patients compared to controls [*t*(45) = −2.49, *P* = 0.04]. The learning problems subscale showed significant group differences [*F*(2,46) = 7.48, *P* < 0.01]. Results were seen in greater problems in the low volume group compared to controls [*t*(46) = −3.6, *P* < 0.01] and the normal volume group compared to controls [*t*(46) = −2.94, *P* = 0.01].

Carer report of fatigue differed across groups [*F*(2,48) = 11.03, *P* < 0.001]. The result of patients in the low volume group having higher levels of fatigue compared to controls [*t*(48) = −4, *P* < 0.001] and the normal volume TBI group also having higher fatigue compared to controls [*t*(48) = −4, *P* < 0.001]. There were significant group differences for carer ratings of participant stress across groups [*F*(2,43) = 7.54, *P* < 0.001]. Patients in the low volume TBI group had higher ratings of stress compared to controls [*t*(43) = −3.9, *P* = 0.001]. No difference was seen for the normal volume TBI group [*t*(43) = −2.1, *P* = 0.09]. Significant group differences were seen for carer reports of executive functioning [*F*(2,41) = 8.31, *P* < 0.001]. Patients with low volume were rated to have more executive problems compared to healthy controls [*t*(41) = −4, *P* < 0.001], as were patients with normal volume [*t*(41) = −2,8, *P* = 0.02] (Fig. 8C).

## Discussion

Long-term outcomes are difficult to predict after paediatric TBI and there is often a poor correlation between the presence or absence of focal brain injury and cognitive, emotional and behavioural disability, as we observe in our study. Our results show that evaluating individual age-matched brain volume provides additional information that helps understand the variation in clinical outcomes produced by paediatric TBI. We describe a robust individualized approach to measuring brain volumes in both grey and white matter that addresses a number of important methodological issues. Brain development creates a major challenge for investigating the effect of TBI on brain structure. To address this issue, we use a large normative dataset of >1200 healthy adolescents to calculate age-specific norms for individual level analysis. We show that brain volume abnormalities are common after moderate/severe TBI in adolescence and reduced brain volumes are associated with a range of post-traumatic problems, unlike focal brain lesions. One-third of our patients had volumetric abnormalities. These were most common in white matter tracts (∼30%) and were particularly prominent in midline white matter structures including the corpus callosum. Patients with low volume had more post-traumatic cognitive and behavioural impairments. Brain volume reductions were associated with slower processing speeds, more anger and more depressive symptoms. Although one-third of the patients also had focal brain injuries and ∼60% had microbleeds, these abnormalities did not show more cognitive or behavioural problems than those without.

Brain volumes change substantially and in distinct ways through adolescence in both grey and white matter regions.^22,55^ Intracranial volume increases through adolescence as the head grows. However, relative to intracranial volume, grey and white matter structures show different development trajectories. Our analysis of >1200 healthy adolescents confirms a gradual reduction in grey matter volumes relative to head size. In contrast, white matter volume tends to increase relative to head size.^22,55^ These non-linear changes show substantial variability across grey and white matter regions, which we describe in detail across a number of white matter tracts and cortical brain regions.

Accurately assessing the impact of TBI on adolescent brain structure requires these developmental changes to be factored into the analysis. We control for age effects on brain structure by comparing individual patients to controls of the same age, taken from our large normative dataset, as well as by focusing analysis on specific cortical, subcortical and white matter regions that show distinct age-related profiles of volume change. We control for the effects of focal brain lesions on the estimation of brain volume by removing lesions from the region of interest and iteratively recalculating the control distribution using established methods.^6,49^ In this way, we are able to classify individuals on the basis of brain volumes while addressing biases arising from complex developmental effects on grey and white matter and any distortions produced by focal brain injuries.

Brain volume abnormalities were common in our patients. Widespread reductions in volume were seen when the whole group was compared to our local controls using a voxelwise analysis. Abnormalities were seen in grey and white matter regions and showed similarities to adult moderate/severe TBI,^7^ including the frontal lobes. Reduced volumes were particularly seen in anterior and posterior cingulate cortices and bilateral insulae cortex. Widespread reductions in white matter volume were also seen in a range of white matter tracts, including the corpus callosum and corticospinal tracts, which are also commonly affected in adult TBI.^6,28,54^ Diffuse axonal injury is a common problem after head injuries of various types. It is produced by shear forces acting on the white matter.^56^ This can lead to progressive neurodegeneration, which results in brain atrophy both in the white matter and grey matter.^7^ We have previously shown that areas of white matter affected by diffuse axonal injury, as indicated by DTI, go on to show brain atrophy.^57^ In addition, other indicators of brain injury such as neurofilament light and tau predict subsequent atrophy rates.^58^ Diffuse axonal injury can occur without any focal injury or diffuse vascular injury.^59^ Hence, post-traumatic volume loss can be explained by diffuse axonal injury and subsequent neurodegeneration, and this may be seen in the absence of focal brain injury.

Individual classification showed that one-third of all patients showed abnormally low volume in at least one white matter or grey matter region, after correction for multiple comparisons. Volume estimates are one radiological measure that are informative when added to other information such as the location of focal brain injury, the number and location of microbleeds and detailed neurocognitive assessments. Our work shows a relationship of individualized volume estimates to cognitive functioning. As seen in case study 2, this has the potential to relate spatial effects with specific cognitive problems, such as attention. The location of axonal injury between the anterior caudate and anterior cingulate gyrus and difficulties in attention align closely with group effects reported previously in an adult TBI sample.^54^ However, cognitive and behavioural difficulties following TBI are likely to have a range of causes, including the effects of other post-traumatic pathologies and the interaction with other factors such as psychological functioning, socioeconomic status, impact on family dynamic and access to rehabilitation. In paediatric samples, there is the added complexity of emerging developmental disabilities that may or may not stem from the injury. We show that volume estimates are sufficient to explain some of the variance of cognitive performance in individual cases that can inform case management. This is informative for clinical management, particularly when other radiological features of focal injury are absent. Hence, our work suggests that including volumetric information is likely to improve disease classification.

Focal brain injuries identified using standard MRI and microbleed abnormalities were also common in our patient group but did not completely overlap with the presence of brain volume abnormalities. Importantly, ∼10% of patients with volume abnormalities showed no focal brain injury or microbleeds. In addition, the presence of focal damage and microbleeds was not associated with cognitive, emotional or behavioural abnormalities, demonstrating that measuring brain volumes after paediatric TBI provides additional information about the underlying cause of post-traumatic disabilities that are commonly observed.

Our focus on cross-sectional assessment of post-traumatic brain volume abnormalities addresses an important clinical challenge. Patients are typically assessed clinically with a single MRI scan and judgements about abnormalities must be made in comparison to normative data. However, longitudinal follow-up scanning on the same patients would provide additional information about progressive changes in brain volume. In adult TBI, such follow-up MRI demonstrates a progressive loss of brain volume that supporting the presence of a neurodegenerative process triggered by the injury.^7,9^ The effect of such an injury in the developing brain is unclear, but it is likely to adversely affect the normal process of development.^4^ This would be expected to result in an alteration in the normal trajectory of white and grey matter development, which should be assessed using longitudinal measures of brain volume change such as the Jacobian Determinant.^7^ Some evidence suggests atrophy continues in the sub-acute phase following paediatric injury.^14^ A further experimental consideration is the potential impact of scanner variability on our results. Our TBI patients were scanned on a single scanner and a local control group was assessed on the same scanner. However, the large control cohort was scanned across a number of different scanners. Our analysis approach is robust to variability in T_1_ acquisition seen across scanners included in the analysis. As seen in the results, the variance contributed across different population data samples to volume estimates was minimal (∼2%). The local study controls were tested against the population data from these combined datasets, and all fell within the expected normal population distributions. Further work including new sites or different acquisition parameters should test for potential site effects and explore the complexities of TBI heterogeneity in more detail. With increased data, additional measures could include quantile regression and calculation of site-specific offsets against the population distribution.^60^ Application of this workflow to larger datasets could also investigate the links to clinical outcomes in more detail. These additional analyses may be well suited for large collaborative efforts, such as ENIGMA-Brain Injury, which are coordinating efforts in advancing research in the paediatric population.^61^

From the population data it was clear that sex related differences were present with males having higher overall volume. However, this effect was removed almost entirely when correcting for TICV. Finally, we took a selective approach to the white and grey matter regions assessed that balanced the investigation of important brain regions with the need to limit multiple comparison corrections. The white matter regions of interest were largely those evaluated in our previous DTI work,^59^ which we extended to cover grey matter regions that were commonly affected by TBI and that have been implicated in post-traumatic clinical problems of various types.^7,9,62,63^ The regions included in our analysis will not comprehensively describe the effect of TBI on brain volume and other brain regions are likely to show volumetric change. However, the pipeline we describe can be adapted to include other white and grey matter regions and future work could usefully extend our analysis using other regions of interest.

In summary, we show how brain volume abnormalities after paediatric TBI can be robustly calculated on an individual basis from T_1_ MRI by generating age-specific estimates from a large normative dataset. This new individualized approach addresses significant methodological issues assessing effects of injury on brain volume in adolescents due to the large variance contributed by age. We also show how these individualized assessments of volume can provide some understanding of related cognitive function following TBI. Alongside other clinical tools, age-specific volume estimates can aid the clinical picture of an injury to better understand the individual case.

## Supplementary Material

awac130_Supplementary_Data

## Abbreviations

TBI: traumatic brain injury
TICV: total intracranial volume
