# Supplementary material for: Brain volume abnormalities and clinical outcomes following paediatric traumatic brain injury
Source: Brain. 2022 Jun 27;145(8):2920–34. doi: 10.1093/brain/awac130 (PMC9420021; doi:10.1093/brain/awac130)
Supplement: awac130_Supplementary_Data [file awac130_supplementary_data.pdf]

Supplementary figures:

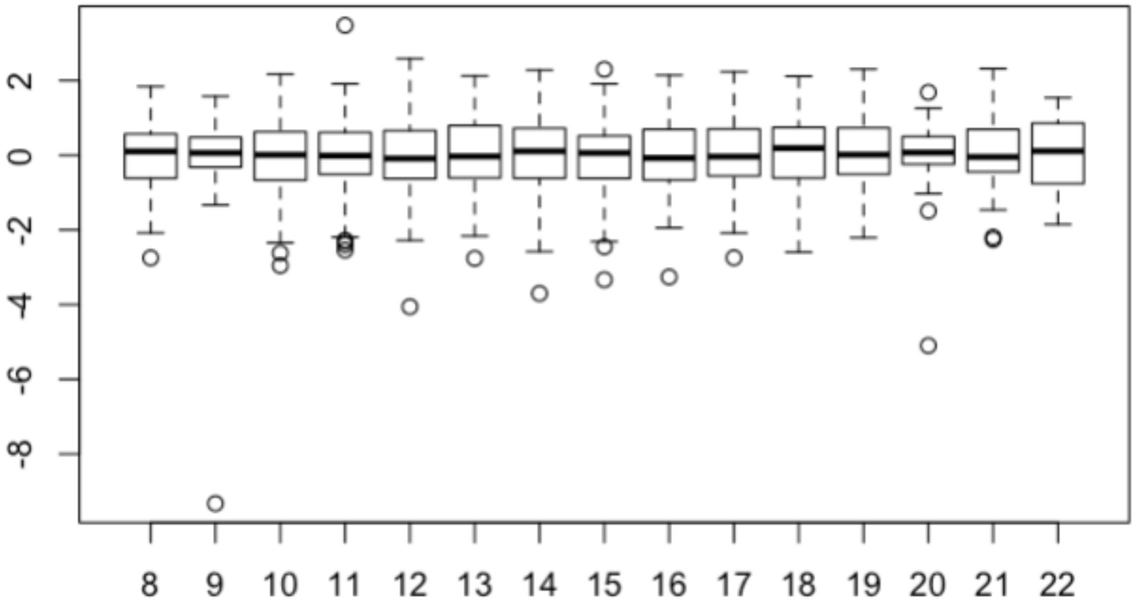

Figure 1: Population data QC for extreme scores. X-axis = age, y-axis = z-score. Data beyond +-3SD were scrubbed from the dataset for further analysis.

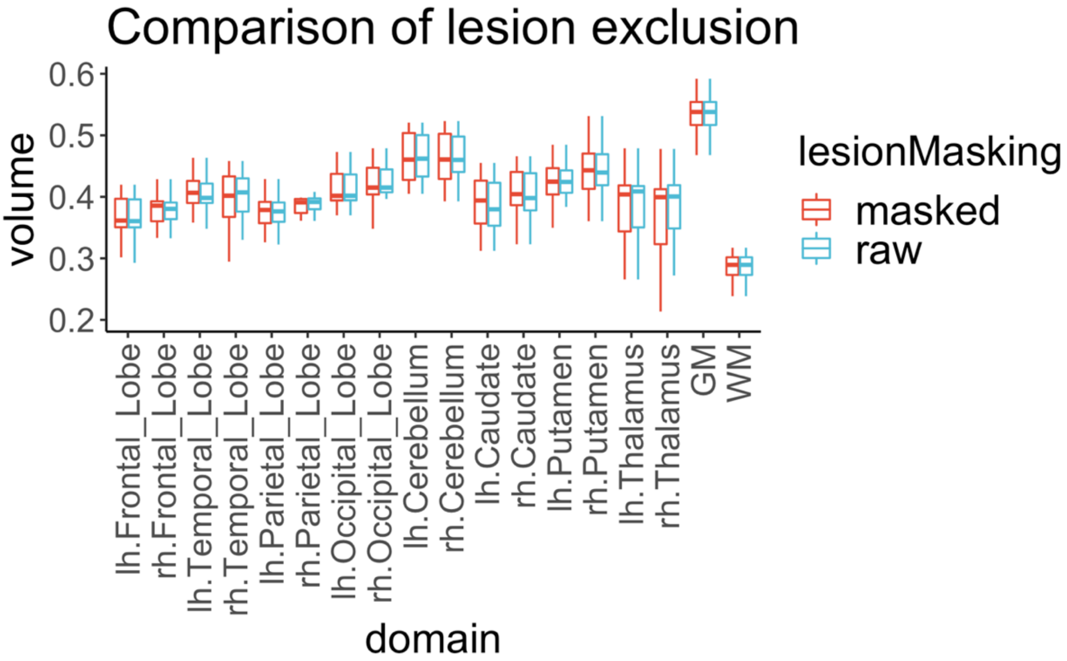

Figure 2: A subsidiary analysis investigating the impact of excluding voxels with focal damage indicating no differences in masking for volume estimates

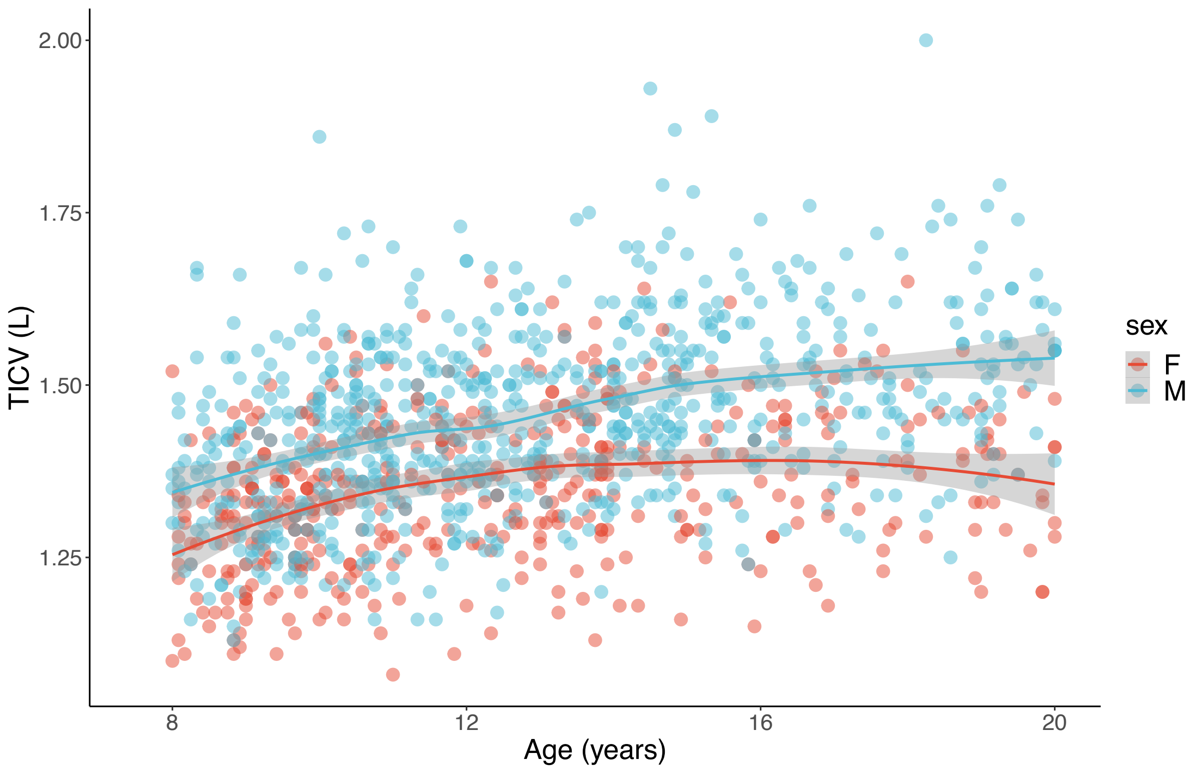

Figure 3: Total Intercranial Volume (L) of population data from 8-22.

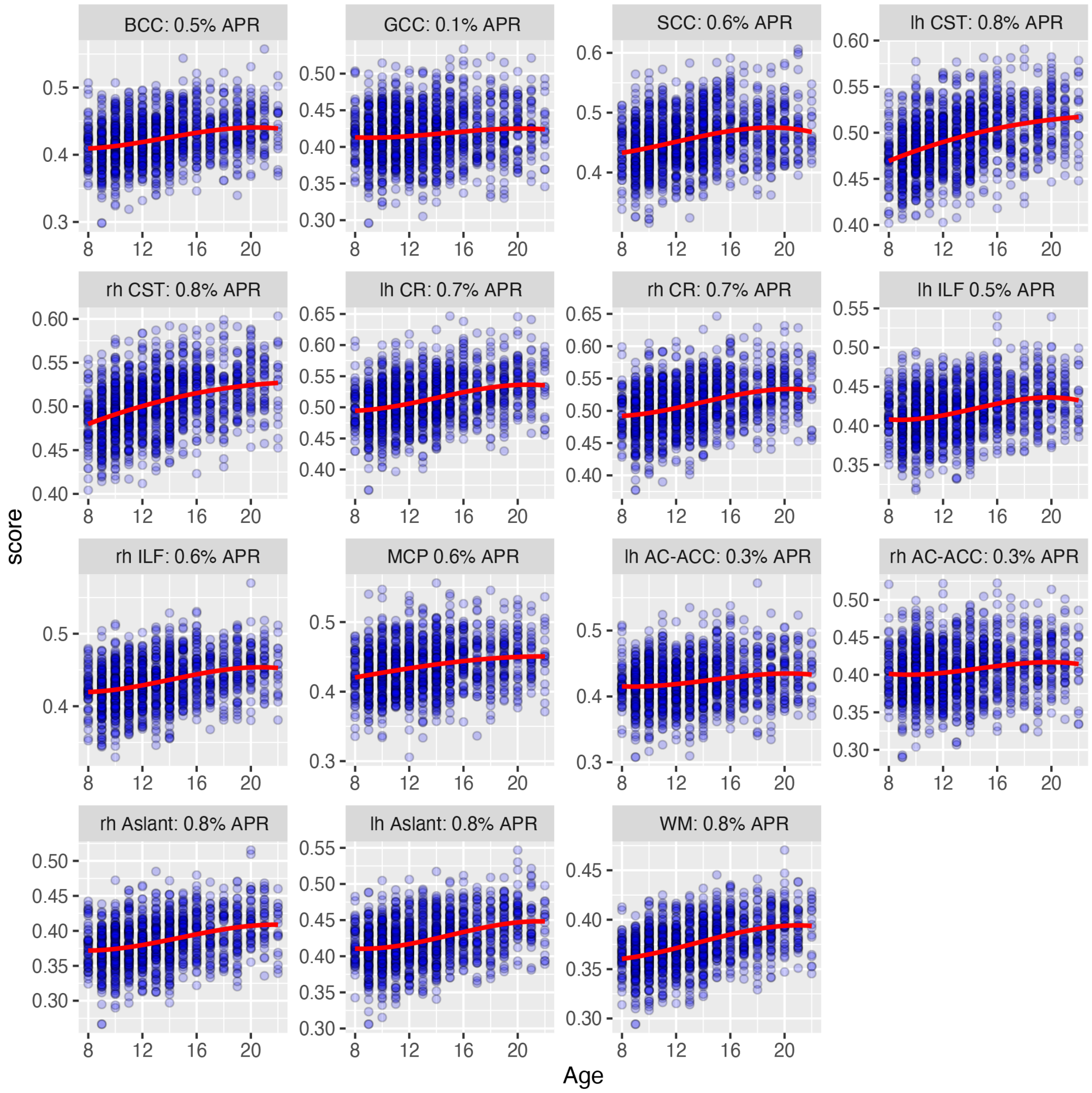

Figure 4: White matter region of interest average annual percentage rate change (APR).

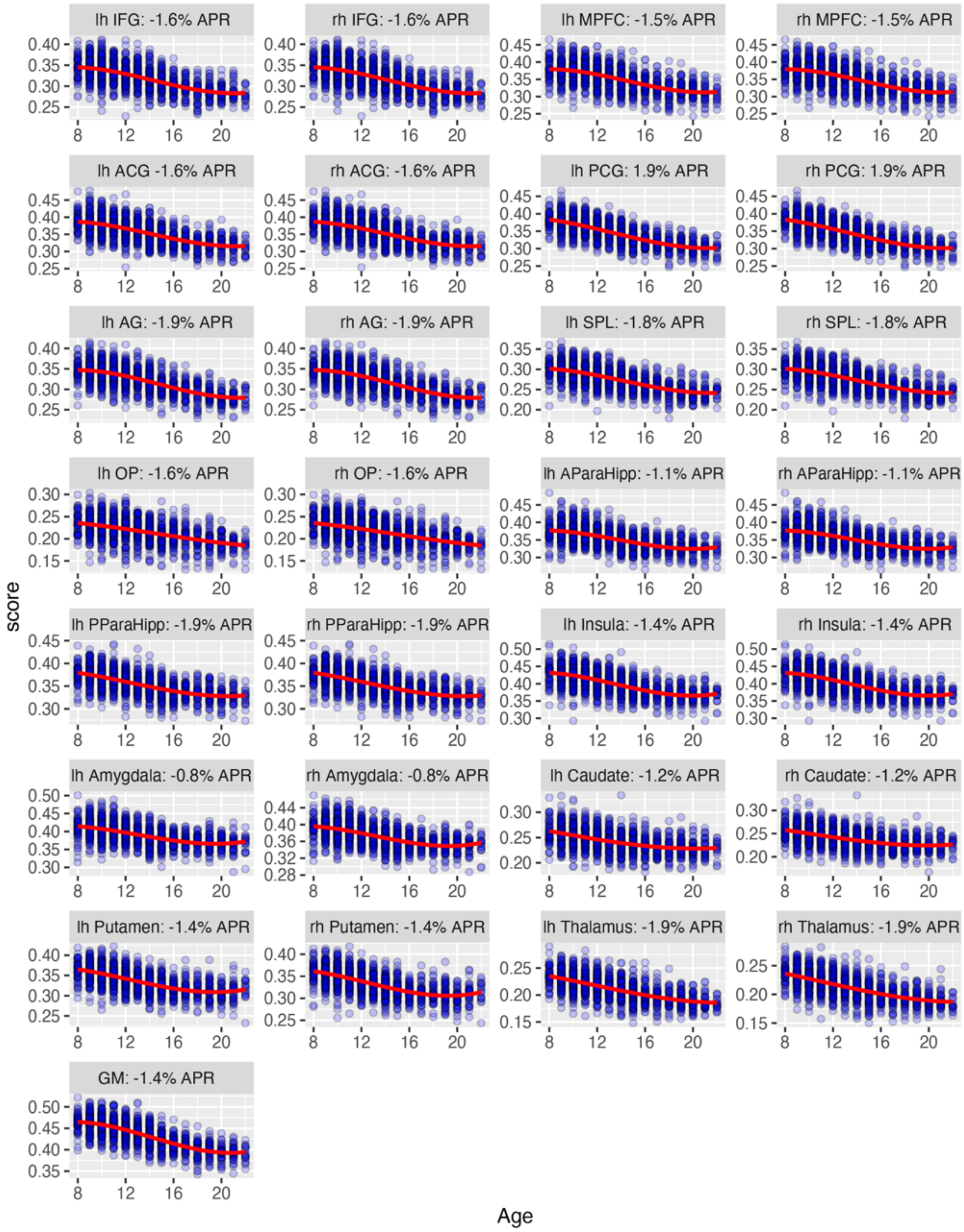

Figure 5: Grey matter ROI age curves

A

Factor	EFA Eigenvalues
1	12.24
2	3.99
3	3.17
4	2.03
5	1.83
6	1.42
7	1.26
8	1.1

B

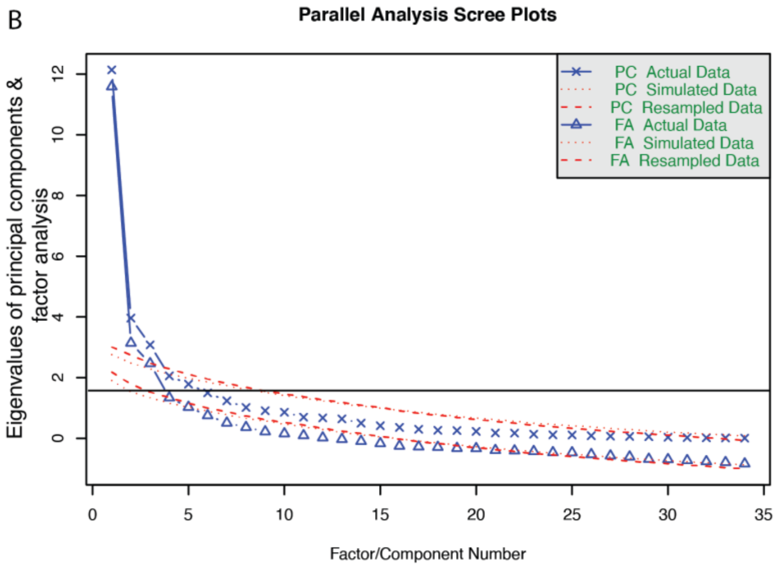

Figure 6: Exploratory factor analysis (EFA). A) Eigenvalues for first 8 factor loadings. B) Parallel scree plot of standardised cognitive measures.

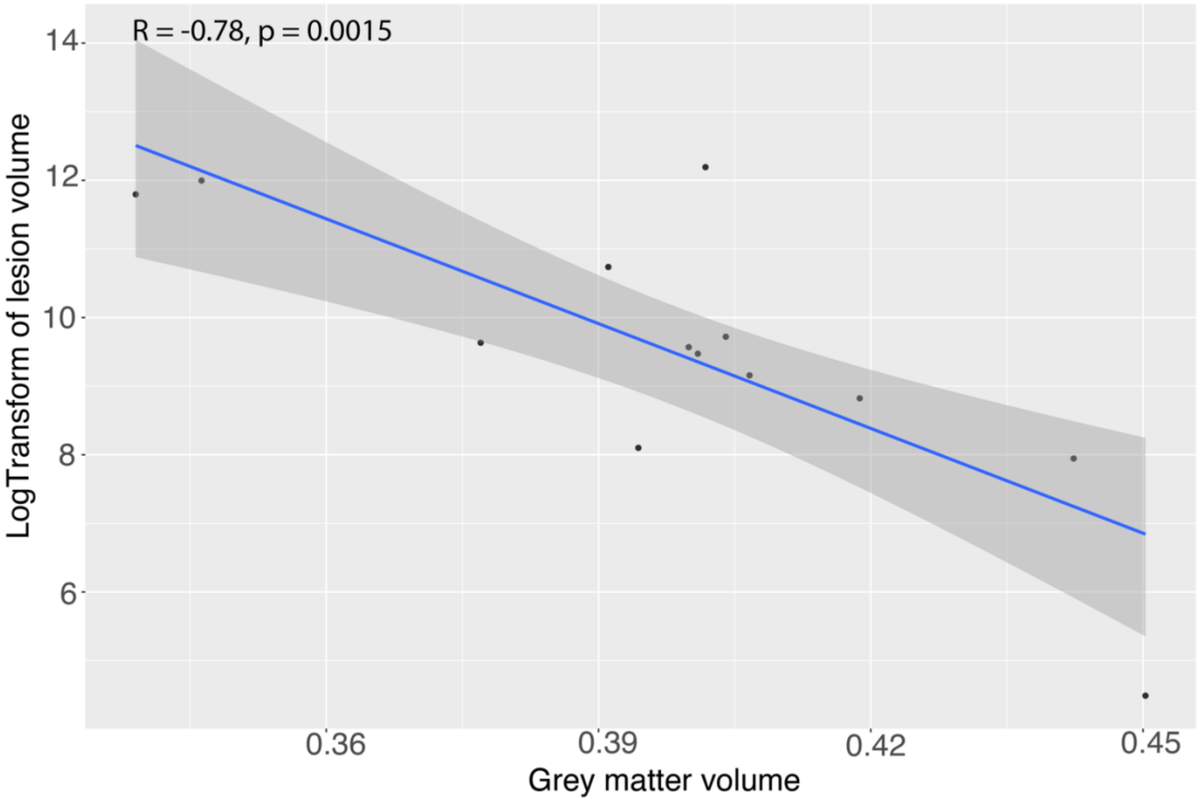

Figure 7: Relationship of log transformed lesion size and brain volume

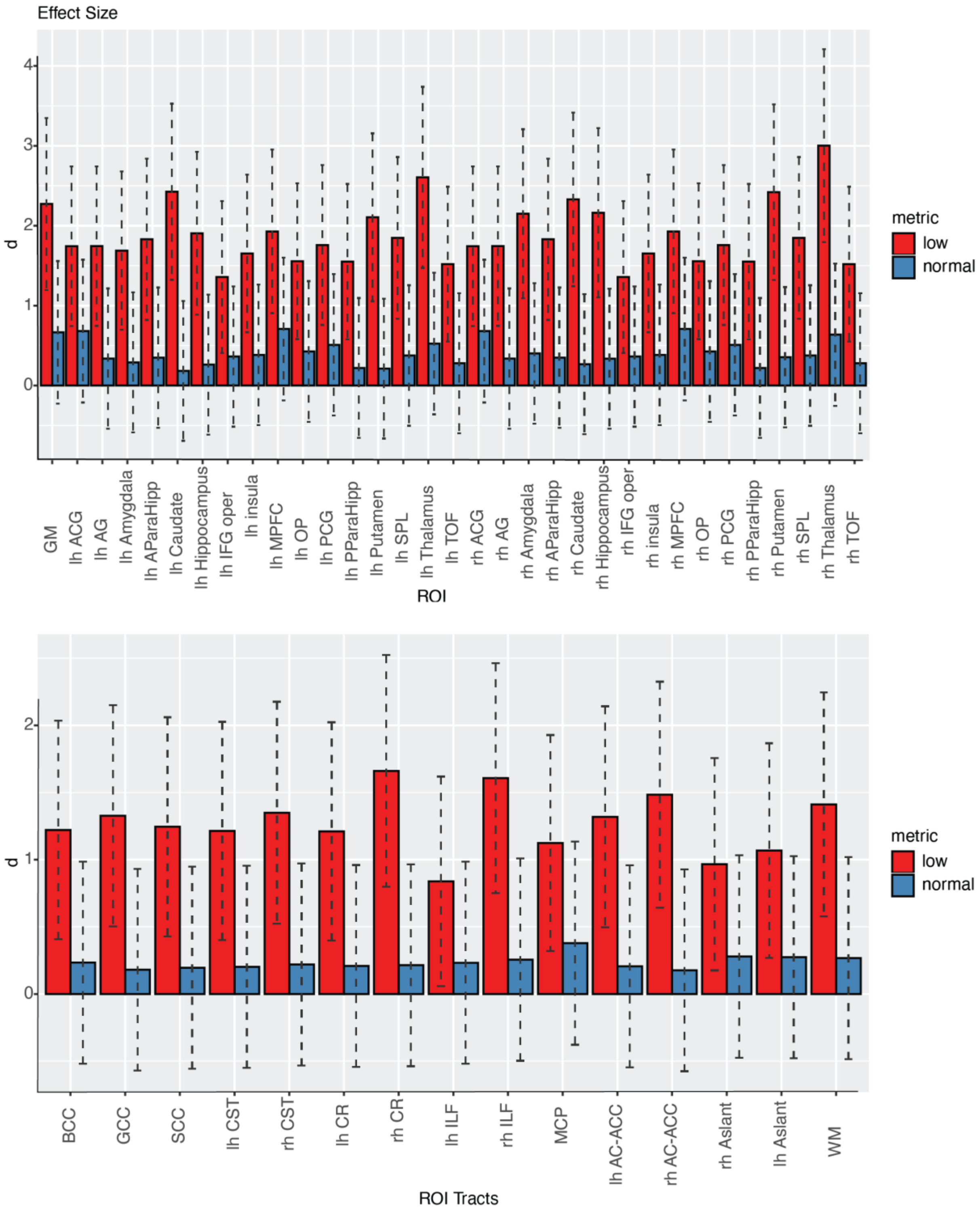

Figure 8: Effect size plot of PTBI patients classified as having low or normal volume in relation to population age norms against local study (Hammersmith) age-matched control

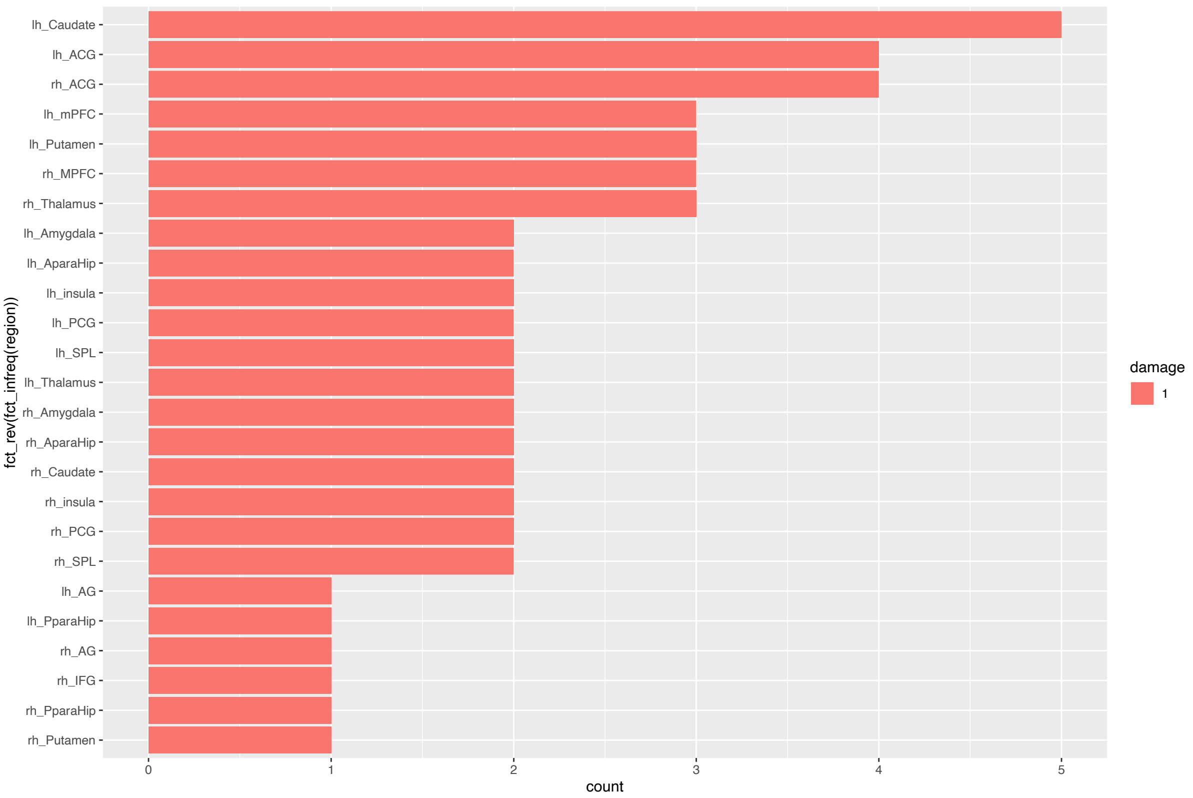

lh_Caudate
lh_ACG
rh_ACG
lh_mPFC
lh_Putamen
rh_MPFC
rh_Thalamus
lh_Amygdala
lh_AparaHip
lh_insula
lh_PCG
lh_SPL
lh_Thalamus
rh_Amygdala
rh_AparaHip
rh_Caudate
rh_insula
rh_PCG
rh_SPL
lh_AG
lh_PparaHip
rh_AG
rh_IFG
rh_PparaHip
rh_Putamen
fct_rev(fct_infreq(region))
0
1
2
3
4
5
count
damage
1

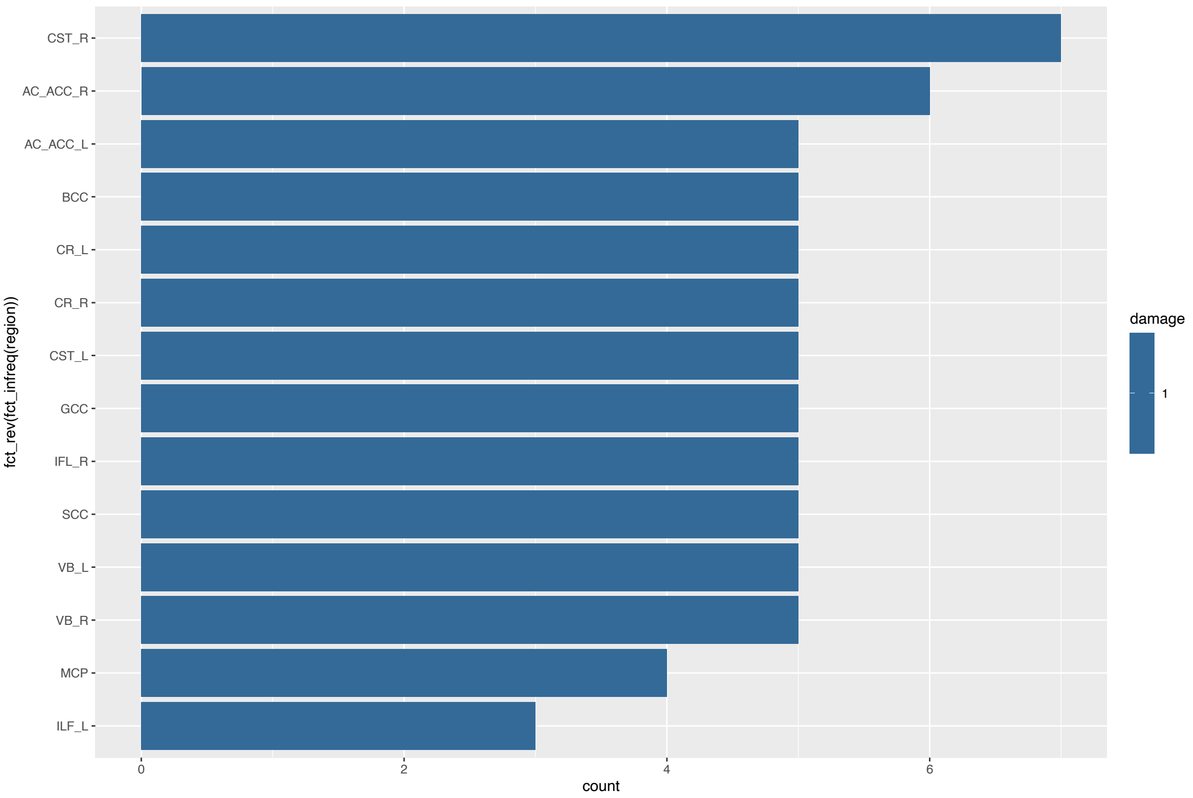

CST_R
AC_ACC_R
AC_ACC_L
BCC
CR_L
CR_R
CST_L
GCC
IFL_R
SCC
VB_L
VB_R
MCP
ILF_L
fct_rev(fct_infreq(region))
0
2
4
6
count
damage
1

Figure 9: ROIs identified with the current pipeline as showing abnormally low volume in the PTBI group in relation to population age norms

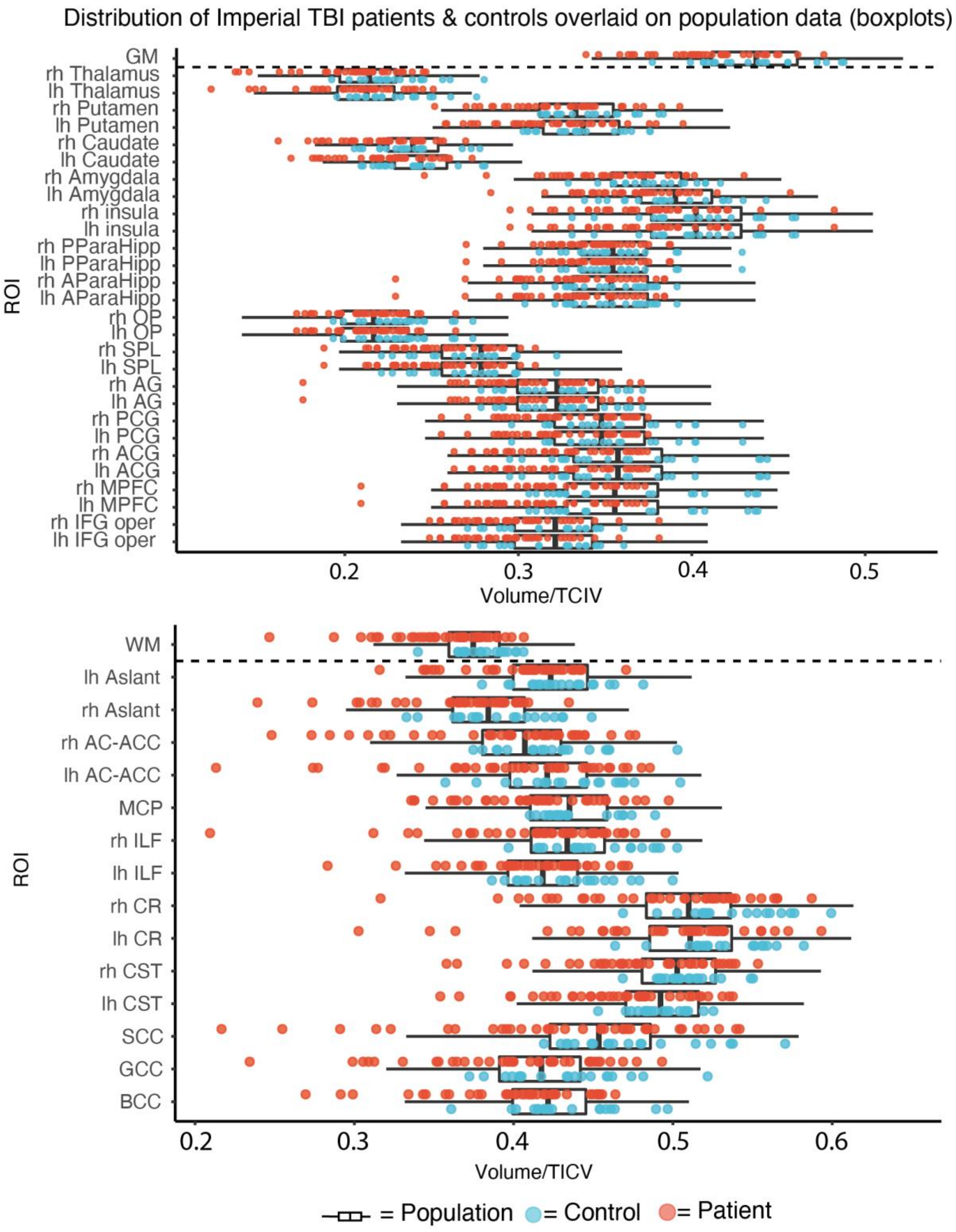

Figure 10: Summary of ROI volume for local study controls (blue) and PTBI patients (red) overlaid on population distributions (boxplots). The local controls sit within the same distribution as the population data. A subset of patients with low volume are

skewed to the left, driving effects in group comparisons with controls. Detailed comparisons for each ROI are provided in supplementary table 7.

Supplementary tables:

Table 1: Demographics for local study participants

		Controls	Patients
**Age at assessment**	Years	13.26 (2.21)	13.54 (1.76)
	Months	164.26 (25.62)	167.54 (21.34)
**Age at Injury**	Years	-	10.55 (4.44)
	Months	-	126.57 (53.14)
**Time since Injury**	Years	-	3.15 (3.67)
	Months	-	38.41 (43.67)

Table 2: Participant clinical characteristics

Participant	Group	Gender	Age at visit (months)	Age at injury (months)	Time since injury (months)	Cause	Mayo Classification	Lowest GCS	LOC Duration
PTBI001	Patient	Female	171	161	9	Accidental/Fall	Moderate-severe	12	13 secs.
PTBI002	Patient	Female	152	143	9	Accidental/Fall	Moderate-severe	15	0
PTBI003	Patient	Male	169	137	32	RTA	Moderate-severe	5	0
PTBI004	Patient	Male	194	165	29	Sport	Moderate-severe	13	0
PTBI005	Patient	Male	183	168	14	RTA	Moderate-severe	15	0
PTBI006	Patient	Male	156	119	36	RTA	Moderate-severe	9	0
PTBI007	Patient	Female	185	179	6	Accidental/Fall	Moderate-severe	3	0
PTBI008	Patient	Male	187	137	49	Sport-Cycle/Fall	Moderate-severe	15	0
PTBI009	Patient	Female	191	157	34	RTA	Moderate-severe	3	Unknown
PTBI010	Patient	Male	191	185	6	Sport	Moderate-severe	15	0
PTBI011	Patient	Male	147	107	40	RTA	Moderate-severe	7	0
PTBI012	Patient	Female	150	144	6	Accidental/Fall	Moderate-severe	15	0
PTBI013	Patient	Male	174	168	6	RTA	Moderate-severe	8	Unknown

PTBI014	Patient	DNC	DNC	DNC	DNC	DNC	DNC	DNC	DNC
PTBI015	Patient	Male	180	144	36	RTA	Moderate-severe	9	Unknown
PTBI016	Patient	Female	185	179	6	Accidental/Fall	Moderate-severe	Unknown	0
PTBI017	Patient	Male	164	2	161	RTA	Moderate-severe	11	0
PTBI018	Patient	Female	150	137	13	RTA	Moderate-severe	9	Unknown
PTBI019	Patient	Male	172	3	168	Violent/Assault	Moderate-severe	Unknown	0
PTBI020	Patient	Male	128	116	12	RTA	Moderate-severe	6	3 minutes
PTBI021	Patient	Male	183	91	92	RTA	Moderate-severe	3	Unknown
PTBI022	Patient	Male	157	84	73	Accidental/Fall	Moderate-severe	9	Unknown
PTBI023	Patient	Male	155	35	120	Impact	Moderate-severe	3	3 hours
PTBI024	Patient	Male	203	191	11	RTA	Moderate-severe	15	0
PTBI025	Patient	Male	129	12	117	Accidental/Fall	Moderate-severe	3	20 minutes
PTBI026	Patient	Female	137	123	13	RTA	Moderate-severe	3	10 minutes
PTBI027	Patient	Female	136	92	43	RTA	Moderate-severe	3	> 3 hours
PTBI028	Patient	Male	197	187	9	Sport-Cycle/Fall	Moderate-severe	10	0
PTBI029	Patient	Male	197	188	9	Deliberate self-harm	Moderate-severe	3	> 3 hours

PTBI030	Patient	Male	157	142	14	RTA	Moderate-severe	6	15 mins.
PTBI031	Patient	Male	133	127	5	Accidental/Fall	Moderate-severe	15	0
PTBI032	Patient	Male	155	146	9	RTA	Moderate-severe	10	Unknown
PTBI033	Patient	Male	173	164	8	Sport	Moderate-severe	6	> 3 hours
PTBI034	Patient	Female	176	162	14	RTA	Moderate-severe	8	0
PTBI035	Patient	Female	187	173	13	RTA	Moderate-severe	8	0
PTBI036	Patient	Male	182	133	49	RTA	Moderate-severe	7	0
PTBI037	Patient	Female	164	160	4	Accidental/Fall	Moderate-severe	13	0
PTBI038	Patient	Male	147	134	13	Accidental/Fall	Moderate-severe	Unknown	0
PTBI039	Patient	Male	136	12	124	Accidental/Fall	Moderate-severe	Unknown	Unknown
PTBI040	Patient	Female	145	74	71	Impact	Moderate-severe	3	> 3 hours
PTBI041	Patient	Male	190	140	49	RTA	Moderate-severe	12	30 secs.
PTBI042	Patient	Female	194	150	43	RTA	Moderate-severe	15	0
PTBI101	Control	Female	159	NA	NA	NA	NA	NA	NA
PTBI102	Control	Male	192	NA	NA	NA	NA	NA	NA
PTBI103	Control	Male	184	NA	NA	NA	NA	NA	NA
PTBI104	Control	Male	181	NA	NA	NA	NA	NA	NA

PTBI105	Control	Male	161	NA	NA	NA	NA	NA	NA
PTBI106	Control	Male	185	NA	NA	NA	NA	NA	NA
PTBI107	Control	Female	203	NA	NA	NA	NA	NA	NA
PTBI108	Control	Female	196	NA	NA	NA	NA	NA	NA
PTBI109	Control	Female	181	NA	NA	NA	NA	NA	NA
PTBI110	Control	Male	141	NA	NA	NA	NA	NA	NA
PTBI111	Control	Female	125	NA	NA	NA	NA	NA	NA
PTBI112	Control	Female	152	NA	NA	NA	NA	NA	NA
PTBI113	Control	Female	149	NA	NA	NA	NA	NA	NA
PTBI114	Control	Male	160	NA	NA	NA	NA	NA	NA
PTBI115	Control	Male	150	NA	NA	NA	NA	NA	NA
PTBI117	Control	Male	166	NA	NA	NA	NA	NA	NA
PTBI118	Control	Female	137	NA	NA	NA	NA	NA	NA
PTBI119	Control	Female	195	NA	NA	NA	NA	NA	NA
PTBI120	Control	Male	124	NA	NA	NA	NA	NA	NA
PTBI121	Control	Female	128	NA	NA	NA	NA	NA	NA

Table 3: Population data table. Top row = age; Bottom row = n

**Age**	8	9	10	11	12	13	14	15	16	17	18	19	20	21	22
**n**	52	131	163	123	128	95	128	83	66	50	44	55	44	32	18

Table 4: Neuropsychology measures. Adjusted with FDR correction for multiple comparisons

**Domain**	**Measure**	**Control mean**	**Control std**	**Patient mean**	**Patient std**	**t**	**p-val**	**q-val (pAdj)**
**IQ**	WASI2 Blocks	0.089	0.844	-0.480	1.196	2.005	0.051	0.074
	WASI2 MatrixR	0.761	0.756	0.069	1.232	2.527	0.015	0.031
	WASI2 Vocab	1.072	0.729	0.224	1.031	3.443	0.001	0.008
	WASI2 Sims	0.778	1.071	-0.165	0.905	3.181	0.003	0.014
	WASI2 VCI	1.004	0.999	0.016	0.942	3.460	0.002	0.008
	WASI2 PRI	0.459	0.840	-0.116	1.026	2.187	0.035	0.060
	WASI2 FSIQ	0.815	0.840	-0.037	0.984	3.276	0.002	0.010
	WISC5 DigitS	0.431	1.033	-0.167	1.077	1.922	0.063	0.086

	WISC5 Coding	-0.020	1.083	-0.902	1.159	2.678	0.011	0.029
	WISC5 SymbolS	0.196	0.951	-0.559	0.945	2.678	0.012	0.029
	WISC5 PSI	0.106	1.115	-0.820	1.091	2.814	0.008	0.028
	WIAT2 WordR	0.263	1.824	0.014	0.732	0.556	0.584	0.584
**Processing speed**	DKEFS1	-0.196	0.972	-0.627	0.840	1.561	0.130	0.154
	DKEFS2	0.216	0.799	-0.362	0.785	2.459	0.020	0.037
	DKEFS 1+2	0.078	0.759	-0.400	0.727	2.161	0.039	0.061
	DKEFS3	0.098	1.235	-0.794	0.998	2.586	0.015	0.031
	DKEFS4	0.235	0.970	-0.600	1.114	2.772	0.009	0.028
	CPT2 Overall RT	-0.242	0.946	0.003	0.878	-0.844	0.406	0.429
	Trails A	29.089	8.667	42.161	16.551	-3.701	0.001	0.005
	Trails B	74.530	29.199	102.188	46.733	-2.586	0.013	0.031
**Executive function**	DKEFS 3-1	0.294	0.790	-0.147	0.972	1.738	0.090	0.118
	DKEFS 4-(1+2)	0.157	0.708	-0.200	0.871	1.577	0.123	0.151
	Trails B-A	45.441	23.775	60.027	38.363	-1.667	0.102	0.129

**Attention**	CPT2 Detectability	0.112	0.776	0.642	0.620	-2.314	0.030	0.054
	CPT2 CRT	-0.277	0.365	0.039	1.037	-1.513	0.138	0.154
	CPT2 Consistency	-0.141	0.481	0.032	1.192	-0.698	0.489	0.502
**Memory**	CVLTC Learning	0.250	1.067	-1.127	1.985	3.343	0.002	0.008
	CVLTC ShortD FreeR	0.167	1.043	-0.492	1.333	1.935	0.060	0.084
	CVLTC ShortD CuedR	-0.056	0.998	-0.406	1.335	1.052	0.298	0.324
	CVLTC LongD FreeR	0.333	1.015	-0.391	1.366	2.130	0.039	0.061
	CVLTC LongD CuedR	0.028	0.915	-0.453	1.316	1.516	0.136	0.154
	CVLTC Recog	0.417	0.462	0.031	0.879	2.031	0.048	0.073
	CMS Stories Imm	1.074	0.890	-0.069	1.219	3.859	0.000	0.005
	CMS Stories Del	1.093	0.948	-0.147	1.242	4.014	0.000	0.004
	CMS Stories Recog	0.963	0.847	-0.461	1.035	5.330	0.000	0.000
	DP People Imm raw	29.889	5.246	25.387	7.168	2.522	0.015	0.031
	DP People Del raw	10.278	2.244	8.129	3.106	2.795	0.008	0.028
	DP Names Total raw	20.833	1.917	18.920	2.798	2.660	0.011	0.029

Table 5: Reports of everyday functioning and wellbeing (Parent & self)

Respondent	Domain	measure	Control mean	Control STD	Patient mean	Patient STD	t	p-val	q-val (pAdj)
Parent	Learning difficulties and adaptive skills	ABAS Parent Communication	11.111	1.491	7.886	2.968	5.266	0.000	0.000
		ABAS Parent Functional Academics	12.389	1.975	9.400	3.164	4.216	0.000	0.001
		ABAS Parent Self direction	9.556	2.382	7.265	2.906	3.052	0.004	0.010
		ABAS Parent Leisure	11.556	2.121	8.441	3.711	3.849	0.000	0.001
		ABAS Parent Social	10.944	1.731	8.324	3.052	3.949	0.000	0.001
		ABAS Parent community Use	12.056	2.388	9.971	3.365	2.605	0.012	0.027
		ABAS Parent Home Living	9.444	2.455	7.629	2.819	2.423	0.020	0.042
		ABAS Parent Health Safety	11.167	2.203	8.735	3.387	3.121	0.003	0.008
		ABAS Parent Self care	10.778	1.263	9.029	2.949	2.979	0.005	0.011
		ABAS Parent General Adaptive	106.056	10.519	90.382	15.327	4.338	0.000	0.000
		ABAS Parent Conceptual	105.944	10.056	89.618	15.194	4.635	0.000	0.000
		ABAS Parent Social	109.000	10.890	92.676	16.444	4.281	0.000	0.001

	ABAS Parent Practical	104.222	10.887	92.941	16.385	2.964	0.005	0.011
Attention-deficit/hyperactivity disorder	Connors3 Parent Inattention	52.063	11.773	67.735	18.253	-3.648	0.001	0.003
	Connors3 Parent Hyperactivity Impulsivity	47.813	8.142	63.606	20.251	-3.880	0.000	0.001
	Connors3 Parent Learning Problems	47.250	8.386	62.206	16.058	-4.321	0.000	0.000
	Connors3 Parent Executive Functioning	52.563	13.356	62.794	15.269	-2.411	0.022	0.044
	Connors3 Parent Defiance Aggression	51.313	11.734	61.647	18.922	-2.362	0.023	0.044
	Connors3 Parent Peer Relations	52.750	14.808	66.088	18.387	-2.743	0.009	0.021
Executive function	BRIEF Parent Inhibit	44.692	6.143	62.281	19.464	-4.581	0.000	0.000
	BRIEF Parent Shift	45.923	8.827	59.000	18.422	-3.210	0.003	0.007
	BRIEF Parent Emotional Control	47.769	8.983	64.313	19.278	-3.919	0.000	0.001
	BRIEF Parent Behavioural Regulation Index	45.615	7.089	63.438	19.614	-4.471	0.000	0.000
	BRIEF Parent Global Executive Composite	47.077	8.789	64.219	16.366	-4.531	0.000	0.000

	BRIEF Parent Initiate	46.692	8.606	62.531	13.481	-4.696	0.000	0.000
	BRIEF Parent Working memory	48.538	7.709	67.094	16.728	-5.085	0.000	0.000
	BRIEF Parent Plan Organise	49.615	10.079	61.531	14.984	-3.094	0.004	0.010
	BRIEF Parent Organisation of materials	48.000	11.554	57.125	14.712	-2.211	0.035	0.066
	BRIEF Parent Monitor	48.077	9.233	59.281	12.311	-3.334	0.002	0.007
	BRIEF Parent Metacognition Index	48.231	9.808	63.250	14.587	-4.007	0.000	0.001
Strengths and difficulties	SDQ Parent Overall Stress	8.067	4.383	15.219	9.054	-3.649	0.001	0.003
questionnaire	SDQ Parent Emotional Distress	2.467	3.067	3.529	2.620	-1.167	0.255	0.407
Mental health	SDQ Parent Behavioural Diffs	1.467	1.457	3.143	2.463	-2.987	0.005	0.011
	SDQ Parent Hyperactivity Attention diffs	3.133	2.167	5.800	2.753	-3.665	0.001	0.003
	SDQ Parent Diffs getting along with other children	1.667	1.839	2.971	2.875	-1.921	0.062	0.108
	SDQ Parent Kind Helpful Behaviour	8.600	1.502	7.265	2.391	2.366	0.023	0.044
	SDQ Parent Impact on life	0.733	1.223	3.941	3.550	-4.677	0.000	0.000

		Children s Memory Q Parent negative worse	0.811	0.345	-1.170	1.634	6.678	0.000	0.000
	Fatigue	PedsQL Fatigue Parent General Fatigue	-0.274	0.874	-2.195	1.692	5.397	0.000	0.000
		PedsQL Fatigue Parent Sleep Rest	-0.218	0.778	-1.749	1.418	5.030	0.000	0.000
		PedsQL Fatigue Parent Mental Cognitive	-0.260	1.153	-2.534	1.946	5.283	0.000	0.000
Self	Wellbeing	BYI2 Self Self concept Inventory	47.500	7.098	45.314	8.185	1.007	0.320	0.483
		BYI2 Self Anxiety	49.611	12.054	52.114	11.537	-0.726	0.473	0.640
		BYI2 Self Depression	48.333	9.947	49.429	8.654	-0.396	0.695	0.854
		BYI2 Self Anger	43.667	8.506	50.114	10.698	-2.388	0.022	0.044
		BYI2 Self Disruptive Behaviour	43.944	6.530	47.371	8.370	-1.639	0.109	0.184
	Fatigue	PedsQL Fatigue Self General Fatigue	-0.937	1.851	-2.102	1.357	2.406	0.023	0.044
		PedsQL Fatigue Self Sleep Rest	-0.432	1.230	-1.309	1.046	2.624	0.013	0.028
		PedsQL Fatigue Self Mental Cognitive	-0.605	1.576	-1.961	1.263	3.219	0.003	0.008

Apathy	LARS Total	-32.053	5.572	-18.600	9.589	-6.517	0.000	0.000
	LARS Everyday Productivity	-3.421	1.017	-2.057	2.155	-3.153	0.003	0.007
	LARS Interests	-3.632	0.831	-2.229	1.926	-3.719	0.001	0.002
	LARS Taking the initiative	-3.316	1.376	-0.343	2.326	-5.896	0.000	0.000
	LARS Novelty Seeking	-3.842	0.375	-2.771	1.832	-3.331	0.002	0.006
	LARS Motivation	-3.579	1.017	-2.086	2.120	-3.492	0.001	0.003
	LARS Emotional Response	-3.368	0.955	-2.229	1.352	-3.600	0.001	0.003
	LARS Concern	-3.316	0.820	-2.086	1.772	-3.478	0.001	0.003
	LARS Social Life	-3.947	0.229	-2.543	1.669	-4.895	0.000	0.000
	LARS Self awareness	-3.632	0.597	-2.057	2.235	-3.917	0.000	0.001

Table 6: Exploratory Factor Loadings

Loadings:	[,1] Memory/learning	[,2] IQ	[,3] RT-1	[,4] Executive	[,5] Attention
WASI2_Blocks_zscore		0.851			
WASI2_MatrixR_zscore		0.854			
WASI2_Vocab_zscore		0.741			
WASI2_Sims_zscore		0.748			
WASI2_VCI_zscore		0.792			
WASI2_PRI_zscore		0.722			
WASI2_FSIQ_zscore		0.909			
DKEFS1_zscore			0.884		
DKEFS2_zscore			0.784		
DKEFS3_zscore			0.467		
DKEFS4_zscore			0.551	0.466	
DKEFS3_minus_1_zscore			-0.304	0.457	-0.376
DKEFS1_plus_2_zscore			0.986		
DKEFS4_minus_1plus2_zscore				0.671	
WISC5_DigitS_zscore		0.377	0.341		
CPT2_OverallRT_zscore					0.483
CPT2_Detectability_zscore				-0.386	0.353
CPT2_ChangeRT_zscore					0.798
CPT2_Consistency_zscore		-0.318			0.596
WISC5_Coding_zscore			0.328	0.565	
WISC5_SymbolS_zscore			0.314	0.503	
WISC5_PSI_zscore			0.34	0.584	
TrailsA_Raw			-0.551	-0.331	
TrailsB_Raw	-0.47		-0.305		

TrailsBminusA_raw	-0.513			
CVLTC_Learning_zscores	0.868			
CVLTC_ShortD_FreeR_zscore	0.754			
CVLTC_ShortD_CuedR_zscore	0.953			
CVLTC_LongD_FreeR_zscore	0.962			
CVLTC_LongD_CuedR_zscore	0.959			
CVLTC_Recog_zScore	0.583			-0.359
CMS_Stories_Imm_zscore	0.552	0.325		
CMS_Stories_Del_zscore	0.633			
CMS_Stories_Recog_zscore	0.625			
DP_People_Imm_raw	0.449		0.331	
DP_People_Del_raw	0.501			
DP_Names_Total_raw	0.383			
WIAT2_WordR_zscore		0.448	0.352	

Table 7. ROI volume estimates between controls and patients classified as having normal or low volume for their age. Analysis of variance (ANOVA) was conducted with Tukey multiple comparison correction between all pairwise means.

Tissue	ROI	Healthy Controls Mean (+/- SD)	TBI: Low Mean (+/- SD)	TBI: Normal Mean (+/- SD)	Low vs. Control	Normal vs. Control	Low vs. Normal
**White matter**	BCC	0.59	0.49	0.58	0.003	0.69	0.01
	SCC	0.56	0.5	0.63	<0.001	0.62	<0.001
	GCC	0.58	0.48	0.58	<0.001	0.75	0.001
	CR L	0.73	0.61	0.61	<0.001	0.64	0.001
	CR R	0.74	0.60	0.60	<0.001	0.62	<0.001
	lh CST	0.68	0.60	0.60	0.01	0.69	0.02
	rh CST	0.7	0.61	0.61	0.005	0.68	0.02
	lh ILF	0.6	0.54	0.54	<0.001	0.65	<0.001
	rh ILF	0.62	0.50	0.50	<0.001	0.66	<0.001
	MCP	0.6	0.54	0.54	0.07	0.37	0.46
	lh Aslant	0.59	0.53	0.53	0.06	0.65	0.22
	rh Aslant	0.54	0.47	0.49	0.03	0.66	0.14
	lh Caudate-ACC	0.6	0.48	0.48	<0.001	0.72	<0.001
	rh Caudate ACC	0.58	0.47	0.47	<0.001	0.75	<0.001
	WM	0.52	0.45	0.51	0.02	0.75	0.08

**Grey matter**	lh IFG	0.44	0.38	0.42	0.01	0.33	0.12
	rh IFG	0.44	0.38	0.42	0.02	0.33	0.13
	lh ACG	0.50	0.40	0.46	<0.001	0.002	0.008
	rh ACG	0.50	0.40	0.46	<0.001	0.002	0.009
	lh PCG	0.49	0.40	0.46	<0.001	0.09	0.01
	rh PCG	0.49	0.40	0.46	<0.001	0.09	0.01
	lh A Para Hipp	0.49	0.41	0.47	<0.001	0.36	0.005
	rh A Para Hipp	0.49	0.41	0.47	<0.001	0.36	0.005
	lh P Para Hipp	0.49	0.43	0.48	0.007	0.68	0.02
	rh P Para Hipp	0.49	0.43	0.48	0.007	0.68	0.02
	lh Insula	0.56	0.46	0.53	<0.001	0.15	0.001
	rh Insula	0.56	0.46	0.53	<0.001	0.15	0.001
	lh Amygdala	0.54	0.45	0.52	0.001	0.37	0.002
	rh Amygdala	0.52	0.42	0.50	<0.001	0.23	0.003
	lh Caudate	0.33	0.25	0.32	0.001	0.86	0.003
	rh Caudate	0.33	0.26	0.32	0.004	0.74	0.01
	lh Putamen	0.46	0.38	0.46	<0.001	0.81	<0.001
	rh Putamen	0.47	0.39	0.46	<0.001	0.39	<0.001
	lh Thalamus	0.31	0.22	0.30	<0.001	0.39	<0.001
	rh Thalamus	0.32	0.22	0.30	<0.001	0.29	<0.001

GM	0.57	0.50	0.55	<0.001	0.27	<0.001

Table 8: Summary of participant classifications. 1) summary of acute CT reports, 2) Radiological reporting of contusion and microbleeds from study MRI, 3) Classification of expected white and grey matter volume for age, 4) Standard scores of selected cognitive domains (Average = 25th-75th percentile, Low = <10th %ile).

Participant	Acute CT	Contusion MRI	Microbleed MRI	WM volume	GM volume	IQ	Executive Functioning	Processing Speed	Memory	Attention
PTBI001	SDH, Contusion	positive	positive	normal	normal	**High**	Average	Average	Average	Average
PTBI002	Contusion	positive	0	normal	normal	Average	Average	Average	Average	Average
PTBI003	Frontal skull fracture	0	0	low	normal	Above average	Average	Average	Average	Low
PTBI004	SDH	0	positive	normal	normal	Average	Average	Average	Below Average	Below average
PTBI005	SDH	0	0	normal	normal	Average	Average	Average	Low	Average
PTBI007	SDH, MLS, Contusion	positive	positive	normal	normal	Above average	Average	Average	Average	Below average
PTBI009	DAI	0	positive	low	low	Below average	Low	Low	Average	Average
PTBI010	EDH	positive	0	normal	normal	Average	Average	Average	Average	Average
PTBI011	Contusion	positive	0	low	normal	Low	Average	Below average	Low	Average
PTBI012	EDH	0	0	normal	normal	Average	Below average	Above average	Average	Average
PTBI013	EDH	positive	positive	normal	normal	Average	Average	Average	Average	Above average
PTBI015	EDH, MLS	positive	positive	low	low	Average	Low	Low	Average	Below average
PTBI017	SDH	0	0	low	normal	Above average	Average	Below average	Average	Average
PTBI018	DAI	0	positive	normal	normal	Below average	Average	Average	Average	Below average
PTBI019	SDH	0	0	low	normal	Above average	Average	Average	Low	Below average
PTBI020	Parietal skull fracture, Oedema	0	positive	normal	normal	Low	Low	Below average	Below Average	NA
PTBI021	DAI	0	positive	normal	normal					
PTBI022	EDH	0	0	normal	normal	Below average	Average	Average	**High**	Average
PTBI023	Contusion	positive	0	normal	normal	Low	Average	Low	Low	**High**

PTBI024	SDH, EDH	0	positive	normal	normal					
PTBI025	SDH	0	0	normal	normal	Average	Average	Average	Average	NA
PTBI026	Contusion	positive	positive	normal	low	Average	Average	Below average	Above average	Average
PTBI027	Unknown	positive	positive	low	low	Above average	Average	Low	Low	Average
PTBI028	Contusion	0	positive	normal	normal	Average	Average	Below average	Average	Average
PTBI029	Skull fracture, SAH	0	positive	normal	low	Average	Average	Low	Above average	**High**
PTBI030	DAI, EDH, SGH	0	positive	normal	normal	Average	Average	Average	Below Average	Above average
PTBI031	EDH	0	0	normal	normal	Above average	Average	Average	**High**	Average
PTBI032	SAH	0	positive	normal	normal	Average	**High**	Low	NA	NA
PTBI033	SAH	positive	positive	low	low	Average	Average	Average	Below Average	Average
PTBI034	Occipital fracture, EDH, SAH	0	positive	normal	normal	Average	Average	Average	Average	Above average
PTBI035	Contusion	0	positive	normal	normal	Average	Average	Average	Low	Above average
PTBI036	Skull fracture, SDH, Contusion	positive	0	low	normal	Average	Average	Average	Average	**High**
PTBI037	Skull fracture, SDH	positive	positive	normal	normal	Average	Below average	Average	Average	Below average
PTBI038	ICH	positive	0	normal	normal	Average	Average	Above average	**High**	Average
PTBI039	Temporal fracture, Contusion	0	0	normal	normal	Below average	**High**	Average	NA	Low
PTBI040	Frontal skull fracture, SDH	0	0	normal	normal	Low	Below average	Low	Low	Average
PTBI041	Contusion	positive	positive	low	normal	Below average	Low	Average	NA	NA
PTBI042	Unknown	0	positive	normal	low	**High**	Average	Average	Average	Average

Legend: DAI = Diffuse Axonal Injury; EDH = Extradural Haematoma; ICH = Intracranial Haemorrhage; MLS = Midline shift; SAH = Subarachnoid Haemorrhage SDH = Subdural Haematoma
